# DMpDP: a Diagnostic Multiple-patient DermoFeature Profile store-and-forward teledermoscopy system

**DOI:** 10.1007/s11517-023-02982-0

**Published:** 2023-12-19

**Authors:** Amira S. Ashour, Basant S. Abd El-Wahab, Maram A. Wahba, Dimitrios I. Fotiadis

**Affiliations:** 1 Department of Electronics and Electrical Communications Engineering, Faculty of Engineering, Tanta University, Tanta, Egypt; 2 Unit of Medical Technology and Intelligent Information Systems, Dept. of Materials Science and Engineering, University of Ioannina, GR 45110 Ioannina, Greece; 3 Department of Biomedical Research, Institute of Molecular Biology and Biotechnology, FORTH, GR 45110 Ioannina, Greece

**Keywords:** Teledermoscopy, Store-and-forward, Patient recorded information, Singular value decomposition

## Abstract

Telehealth demand is rapidly growing along with the necessity of providing wide-scale services covering multiple patients at the same time. In this work, the development of a store-and-forward (SAF) teledermoscopy system was considered. The dermoFeatures profile (DP) was proposed to decrease the size of the original dermoscopy image using its most significant features in the form of a newly generated diagonal alignment to generate a small-sized image DP, which is based on the extraction of a weighted intensity-difference frequency (WIDF) features along with morphological features (MOFs). These DPs were assembled to establish a Diagnostic Multiple-patient DermoFeature Profile (DMpDP). Different arrangements are proposed, namely the *horizontally aligned*, the *diagonal-based*, and the *sequential-based* DMpDPs to support the SAF systems. The DMpDPs are then embedded in a recorded patient-information signal (RPS) using a weight factor *β* to boost the transmitted patient-information signal. The effect of the different transform domains, *β* values, and number of DPs within the DMpDP were investigated in terms of the diagnostic classification accuracy at the receiver based on the extracted DPs, along with the recorded signal quality evaluation metrics of the recovered RPS. The sequential-based DMpDP achieved the highest classification accuracy, under − 5 dB additive white Gaussian noise, with a realized signal-to-noise ratio of 98.79% during the transmission of 248 DPs using *β* = 100, and spectral subtraction filtering.

**Graphical Abstract:**

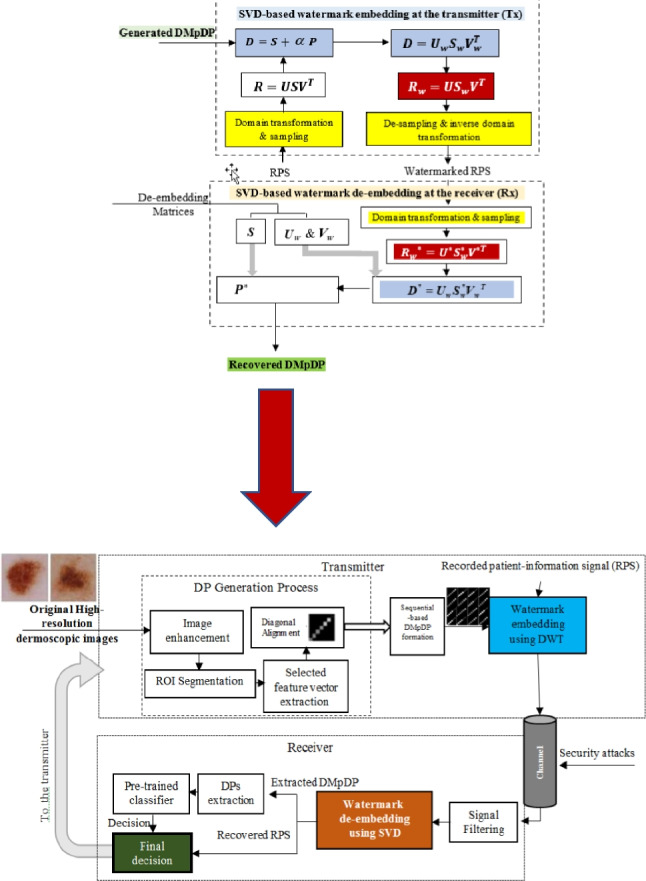

## Introduction 

The COVID 19 pandemic motivated the use of widespread telemedicine and tele-consultation to mitigate waiting lines in hospitals/clinics and to avoid its spreading and infection [1]. Telemedicine changes the communication between physicians, as well as the physicians and their patients. Since skin diseases have a variety of types starting from cancer-related to noncancer-related diseases, the development of a teledermoscopy system is critical to prevent, diagnose, and treat skin diseases remotely without direct interaction [2]. Teledermoscopy is considered an economically feasible choice to achieve telehealth sessions to diagnose skin diseases, including melanocytic lesions leading to optimized care with accurate diagnosis comparable to in-person diagnostic visits [3–6]. Several studies confirmed the superiority of the teledermoscopy diagnostic capacity as opposed to the in-person diagnosis which has the drawback of mishandling melanoma cases [7–9].

Teledermoscopy requires the transmission of the skin disease historical data along with the examination in the form of clinical data and dermoscopic images [10]. The most common teledermoscopy system is based on store-and-forward (SAF) transmission for consultation by sending the dermoscopic image of the unusual skin lesion accompanied by the patient data through a secure web service to an expert [11]. Subsequently, the treating physician retrieves the transmitted images and data and then sends back a diagnosis, treatment guidance, or a therapy plan. Constantly, the SAF teledermoscopy systems allow expert decisions in excessive demand situations or remotely.

Teledermoscopy systems have several requirements for the monitoring of chronic cases with recurrent follow-up and continued treatment, including the capability to transmit multiple images for the same patient captured over several time instances, transmit multiple images for different patients simultaneously, avoid telecommunication channel congestion, reduce delay, and provide protected data transmission. Thus, several requirements and pre-conditions should be considered while realizing a teledermoscopy system, including safety, data encryption, storage, network protection, easy accessibility, and reliable/accurate diagnosis. Also, patients’ recorded information must be provided for guiding the final diagnoses, including patient demographics (e.g., name, age, gender); the patient history (e.g., family history of skin disease, sun exposure, symptoms and complaints, history of tumors, allergies); and the skin lesion’s description (e.g., number of lesions, surface, size, borders, shape, distribution, location, color) [2].

## Related work

To support teledermatology, the efficient storing and self-assessment of skin lesions was introduced based on a smartphone teledermoscopy system [12]. This system acquired, identified, and classified the different skin images into melanoma, benign, and nevus lesions with transmission size reduction. Along with the requirement to preserve the communication channel bandwidth, robust behavior against security attacks is also essential in teledermoscopy systems, which can be achieved using watermarking procedures. Jamali et al. [13] employed the singular value decomposition (SVD) with the discrete cosine transform (DCT) and the discrete wavelet transform (DWT) in embedding a watermark in the medical images. Also, several methods have been proposed for achieving secure transmission in telemedicine systems based on wavelet watermarking [14–17]. However, these methods mainly targeted patient identification and authentication, while other methods targeted tamper detection [18, 19].

On the other hand, Khaldi et al*.* [20] presented a frequency-domain watermarking method for hiding electronic patient records in their electrocardiogram signals. The signal was transformed into a 2D image, and the frequency content of the image was extracted using the integer wavelet transform. Then, Schur decomposition was used to acquire the coefficients, and the watermark bits were integrated by altering the least significant bit of the generated Eigenvalues. Khudhair et al*.* [21] proposed a reversible data hiding technique based on the histogram bin distribution of the cover image, also, the secret data were encrypted before embedding to further strengthen security. Generally, many researchers have proposed different multimedia securing techniques based on watermarking, such as watermarking based on contourlet transform (CT) and singular value decomposition (SVD) [22], chaotic maps [22–25], and range image watermarking [26].

The standardization of the dermoscopic image quality for efficient transmission and reception is considered a key factor in implementing any teledermoscopy system. Arzberger et al. [27] mentioned that images having 800×600 pixel resolution are sufficient, with perfect recommended resolution 1024×768. In the traditional SAF, the digital pictures of skin lesions in the form of the traditional dermoscopy image are transmitted along with the patient’s clinical data to the dermatologist through the intranet or internet using relatively long transmission time and high transmission bandwidth.

The key motivation for the present work is to resolve the transmission limitation due to the large transmission size of dermoscopic images through the traditional SAF system even after compression, especially in the case of transmitting multi-dermoscopic images for the same patient or multiple images for different patients at the same time along with other patient’s information. These scenarios affect the performance of the teledermoscopy systems since the transmission channel bandwidth as well as the image quality are highly affected. In the proposed system, a new framework for the dermoscopic image presentation is engaged to reduce the size of the transmitted dermoscopy image along with the patient’s information, and an innovative representation of the dermoscopic image is proposed, the Diagnostic Multiple-patient DermoFeature Profiles (DMpDP), based on the small unit representing a single dermoscopy image which is called DermoFeature Profile (DP).

Hence, the main highlights covered by this work can be summarized as follows:

- Proposing a novel DP which is an innovative compact form of the original dermoscopy image to substitute the need of transmitting high-resolution dermoscopy images through SAF teledermoscopy systems.
- Presenting a new diagnostic SAF dermoscopic image representation for telemedicine that can be used directly as the main support for the physician in a secure manner.
- Transmitting the patients’ information along with the DP or DMpDP as a recorded patient-information signal, called RPS by embedding the DP or DMpDP in the RPS.
- Collating multiple DPs in different forms composing the DMpDP to represent the medical cases of multiple patients.
- Transmitting the DP or the DMpDP as an embedded watermark in the RPS, which provides integrated, compact and secure representation for the patient(s) case file at the receiver diagnostic side.

Accordingly, the organization of this paper is as follows. Section 2 reports the significant related studies. Then, Sect. 3 introduces the methodology of the proposed SAF teledermoscopy system. In Sect. 4, the results of different experimentation scenarios are reported. In Sect. 5, the proposed system performance is compared to state-of-the-art studies. Finally, the conclusions are presented in Sect. 6.

## Materials and methods of the proposed SAF teledermoscopy system

A dataset of 1400 images equally covering four classes was used. The dataset included benign Keratoses lesions, basal cell carcinoma, melanoma, and melanocytic nevi classes with 350 images per class. The used dataset was obtained from the “ISIC 2018: Skin Lesion Analysis Towards Melanoma Detection” grand challenge datasets [28, 29]. In addition, we synthetically recorded patients’ information through audio signals to simulate the real-time scenarios, where the patient or the physician on the transmitter side records the symptoms, a description of the lesion, and/or any required clinical/ historical data related to the transmitted images. Then, the DP images were embedded in these recorded signals (RPS) for transmission.

The proposed system includes several stages: (i) DP generation including dermoscopy image enhancement, segmentation, feature extraction, and selection to provide an accurate diagnosis, as an expert system, using the generated DMpDP; (ii) DMpDP formation; (iii) DMpDP embedding in the patient-information recorded signal; (iv) filtering; (v) DMpDP extraction from the watermarked signal; (vi) DP extraction; and (vii) classification using a pre-trained classifier. Each of these stages will be explained in the following subsections.

### Generation of proposed Diagnostic Multiple-patient DermoFeature Profile at the transmitter

The DMpDP was generated at the transmitter edge representing the main features of the dermoscopic images under examination. The unit of the DMpDP is the DP, which represents the generated diagonal-aligned features for one patient for a single instance. The generation of a single DP involves the application of sequential image-processing steps to process the original high-resolution dermoscopy image, which includes image enhancement, area-of-interest segmentation, feature extraction and selection, and diagonal representation. For image enhancement, the original/traditional (high resolution) dermoscopy image was preprocessed for artifact removal using DullRazor [30] and median filtering. Subsequently, the lesion was detected using wavelet-based segmentation [31]. Several geometric and color features were extracted from the detected lesion to represent its morphological characteristics [32], in addition to its textural features. The weighted intensity-difference frequency (WIDF) was extracted for the textural representation of the lesion. Those features estimate the smoothness of any given lesion based on the difference in the intensity levels between the pixel pairs inter-sampled at a given distance between the pixels and orientation. For this purpose, the frequency of intensity differences from 0 to *ξ* within the image was estimated, where *ξ* is the number of intensity levels (e.g., *ξ* = 256 for grayscale image). Then, these frequencies were weighted by weights that decrease as we quantify the frequencies of higher intensity differences. Hence, the WIDF at a given inter-sample distance *δ* and a given orientation *θ* is given as:

1 $$WID{F}_{\delta ,\theta }=\frac{\sum\limits_{i=0}^{\xi -1}(\xi -i){f}_{i}}{\xi },$$

where ${f}_{i}$ is the frequency of the intensity difference *i* for the pixels inter-sampled by distance *δ* and orientation *θ* in a given image. Accordingly, the frequencies of the small intensity differences were assigned higher weights compared to the higher intensity differences to describe the textural similarity within the lesion. In addition, morphological features (MOFs) were extracted from the original dermoscopic image. These include the solidity index ${S}_{I}$, the contour steepness ${C}_{T}$, the geometric index ${G}_{I}$, and the pigment deviation ${P}_{D}$ [33]. The solidity index can be calculated using the following equation:

2 $$SI=\frac{P{P}^{2}}{4\Pi {A}_{t}}$$

where *PP* is the lesion perimeter and ${A}_{t}$ is its total area. The contour steepness ${C}_{T}$ is the distance from the centroid to any point on its perimeter. It is based on the different radial distances of the irregular borders of the lesion. ${C}_{T}$ was calculated using the variance of the radial distances by initially determining the location of the binary lesion mask’s centroid. Then, a convolutional 2-D filter was used to scan the lesion for locating the points on the lesion perimeter. For each point on the lesion’s perimeter, the radial distance ${D}_{r}$ was calculated using the following equation:

3 $${D}_{r}=\sqrt{{(a-{c}_{a})}^{2}+{(b-{c}_{b})}^{2}}$$

where ${c}_{a}$ and ${c}_{b}$ are the coordinates of the centroid, while *a* and* b* are the coordinates of the point on the lesion perimeter. Finally, ${C}_{T}$ was calculated as the variance of the obtained radial distances normalized by the mean radial distance*.*

The geometric index indicates the complexity of fractal patterns by measuring how the details change with the scale. Thus, box-counting was used, where the lesion boundary was determined and then covered with a grid to count the number of the occupied boxes at that grid. This process was repeated using a finer grid with smaller boxes until accurately capturing the structure of the pattern. The geometric index can be formulated as follows:

4 $${G}_{I}=\frac{\mathrm{log}({N}_{B})}{\mathrm{log}({L}_{B})}$$

where ${N}_{B}$ is the number of the smallest boxes that covered the edge line, and ${L}_{B}$ is the inverse of the smallest box side length.

The aforementioned MOFs were calculated from the binary lesion mask, while the pigment deviation was calculated from the colored image. The pigment deviation indicates whether the transition between the skin lesion and the surrounding skin is fading slowly or does it have a steep change, which is considered as a warning sign. It was calculated from the mean and variance of the luminance gradient over the lesion’s boundary by converting the RGB segmented lesion into the HSV plane, then measuring the gradient of the *V* channel and calculating its mean ${V}_{M}$ and variance ${V}_{V}$. Diameter, asymmetry, and color features were also extracted [34].

Subsequently, the most discriminative features were selected using the supervised infinite feature selection (Inf-FS) technique [35]. This resulted in the selection of 7 significant features, namely the four WIDF features (${\mathrm{WIDF}}_{\mathrm{10,0}},\mathrm{ WID}{\mathrm{F}}_{\mathrm{10,45}},\mathrm{ WID}{\mathrm{F}}_{\mathrm{10,90}},\mathrm{ WID}{\mathrm{F}}_{\mathrm{10,135}}),$ the diameter, the geometric index, and the contour steepness. These seven features were diagonally aligned to form the single DP.

After features were extracted, watermarking was deployed in the proposed guided SAF teledermoscopy system for hiding the diagnostic profiles that represent the image features in the recorded patient-information signal (RPS). A diagonal alignment of the selected feature vector was proposed to simplify the watermarking extraction process at the receiver using the proposed compacted singular value decomposition-based watermarking. In the present work, multiple DPs were gathered to form the DMpDP, serving many patients in the proposed guided SAF system. Then, the DMpDP was embedded into the RPS before its transmission.

### Compacted singular value decomposition (SVD)-based watermarking

Since SVD is commonly used for watermarking, the singular values are robust against attacks and have high resilience when the image is affected by small perturbations [36]. The SVD watermarking was used in this proposed system, where the generated DMpDP was embedded in the one-dimensional (1D) RPS. Different transform domains were investigated for the watermarking process. Afterward, at the receiver edge, a reverse procedure was implemented to extract the DMpDP from the watermarked recorded signal.

#### DMpDP embedding process

The proposed DMpDP was embedded into the RPS, after converting the recorded signal from the time domain into a frequency domain using discrete cosine transform (DCT), discrete sine transform (DST), or discrete wavelet transform (DWT). In the present work, three forms of DMpDPs were presented, including the horizontally aligned DMpDP, the sequential-based DMpDP, and the diagonal-based DMpDP.

**Fig. 1:**
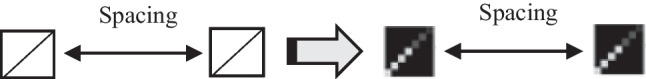
The proposed horizontally aligned DMPDP representation

The first form is the horizontally aligned DMpDP, where the DMpDP is formed using two separated DP for the same patient or different patients (Fig. 1).

Each of the DPs in the horizontally aligned DMpDP was embedded in the recorded signal separately with a pre-determined spacing *μ* between the different DPs. Since the watermark *P* is the 2-D dimensional 7 × 7 2D matrix representing the DP “image” *R*, an RPS segment of 49 samples is required to embed each DP.

**Fig. 2:**
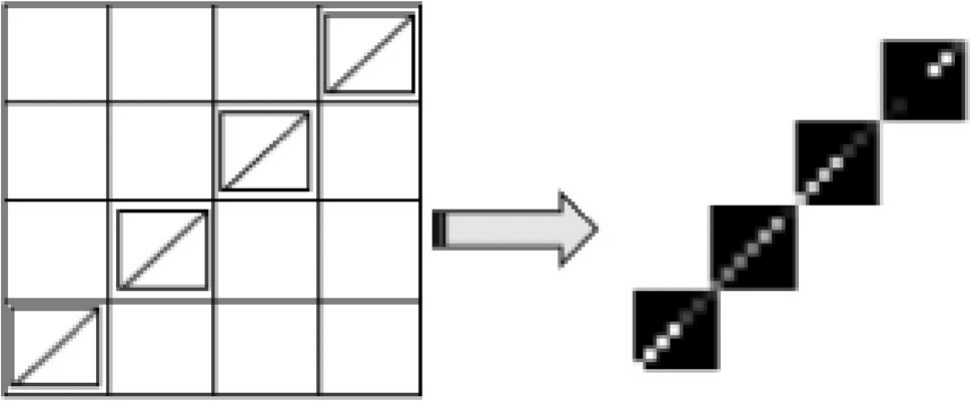
The proposed diagonal-based DMPDP representation

The second form is the diagonal-based DMPDP, where the DMpDP contains *N* different DPs arranged in a sequence of diagonal forms to obtain the diagonal matrix *P* (Fig. 2).

The matrix *P* of the DMpDP was embedded into the recorded signal. Since *P* is two-dimensional with size 7*N* × 7*N*, the required signal segment content for embedding includes $49{N}^{2}$ samples, where this segment was extracted from the 1D transformed recorded signal.

**Fig. 3:**
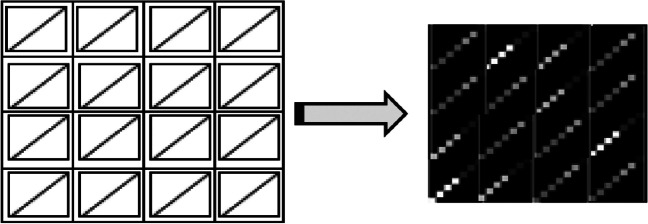
The proposed sequential-based DMPDP representation

The third form is the sequential-based DMPDP, where the DMpDP contains *N* different DPs arranged in a horizontal sequence to obtain the sequential matrix “image” *P*. In this form, the different DP images are arranged sequentially (Fig. 3).

The image *P* of the DMpDP was embedded into the recorded signal. Since the matrix *P* has dimension 7* M* × 7* M*, where *M* = *N*/2, a signal segment of 49*M*^2^ samples is required for embedding.

Accordingly, the size of the generated DMpDP image and the number of the required samples in the recorded signal in the case of the sequential-based DMpDP and the diagonal-based DMpDP is given as:

5 $$L=49{M}^{2},$$

where *M* = *N* in the case of the diagonal-based DMpDP, and *M* = *N*/2 in the case of sequential-based DMpDP, N is the number of DPs. Using the horizontally aligned DMpDP, *M* = *N* and the size of the generated DMpDP image as well as the required number of samples in the recorded signal is as follows:

6 $$L=49M+\mu \left(M-1\right),$$

where *μ* is the distance “spacing” between the DPs, (*μ* = 25 provides the best classification accuracy). From the previous forms of the DMpDP, the recorded speech signal is either utilized in the time domain DST, DCT, or DWT. After that, the recorded signal was divided to take the part in which the number of sample properties with the size of the embedding watermark and converted it from 1 to 2D to obtain *R* matrix. Then, the traditional SVD was applied to the *R* matrix.

7 $${{R}}={{U}}{{S}}{{{V}}}^{{{T}}}$$

where *U*, *S*, and *V* are the left singular, the singular, and the right singular vectors for the *R* matrix, respectively. Where *U*, and *V* are considered as orthogonal matrices. Then, the watermark matrix *P* was inserted to the *S* matrix of the *R* matrix.

8 $${{D}}={{S}}+{\alpha }{{P}},$$

where *α* is the controlling scale factor of the watermark’s strength. Then, the matrix *D* was decomposed using SVD:

9 $${{D}}={{{U}}}_{{{w}}}{{{S}}}_{{{w}}}{{{V}}}_{{{w}}}^{{{T}}},$$

where ${U}_{w}$, ${S}_{w}$, and ${V}_{w}$ are the left singular, the singular, and the right singular vectors for the watermarked matrix D, respectively. Subsequently, the watermarked recorded signal ${\mathrm{R}}_{\mathrm{w}}$ were obtained by utilizing the modified ${S}_{w}$ matrix, as follows:

10 $${{{R}}}_{{{w}}}={{U}}{{{S}}}_{{{w}}}{{{V}}}^{{{T}}}$$

Then, the 2D watermarked recorded signal (${R}_{w}$ matrix) was converted into a 1D vector. The 1D watermarked recorded signal part was returned to its position in the totally transformed recorded signal. Finally, to obtain the transmitted 1D watermarked recorded signals. Then, the inverse transform domain was applied if the recorded signal was performed in the transform domain.

#### De-embedding process of DMpDP

At the receiver edge, the transmitted DMpDP was extracted from the received RPS through the de-embedding process. IF ${U}_{w}$, ${V}_{w},$ and *S* matrices are shared separately as images between the receiver and transmitter sides to extract the DP correctly in the form of ${R}_{w}^{*}$, the transform domain was applied on the received RPS, if the transform domain was accomplished in the embedding process at the transmitter, then converted it from 1 to 2D. Using SVD. Consequently, the SVD was applied in the ${R}_{w}^{*}$ matrix:

11 $${{{{R}}}_{{{w}}}}^{{*}}={{{U}}}^{{*}}{{{S}}}_{{{w}}}^{{*}}{{{V}}}^{{*}{{T}}},$$

12 $${{{D}}}^{{*}}={{{U}}}_{{{w}}}{{{S}}}_{{{w}}}^{{*}}{{{{V}}}_{{{w}}}}^{{{T}}}.$$

The DMpDP is obtained as:

13 $${{{P}}}^{\boldsymbol{*}}=\frac{{{{D}}}^{\boldsymbol{*}}-{{S}}}{\alpha }.$$

**Fig. 4:**
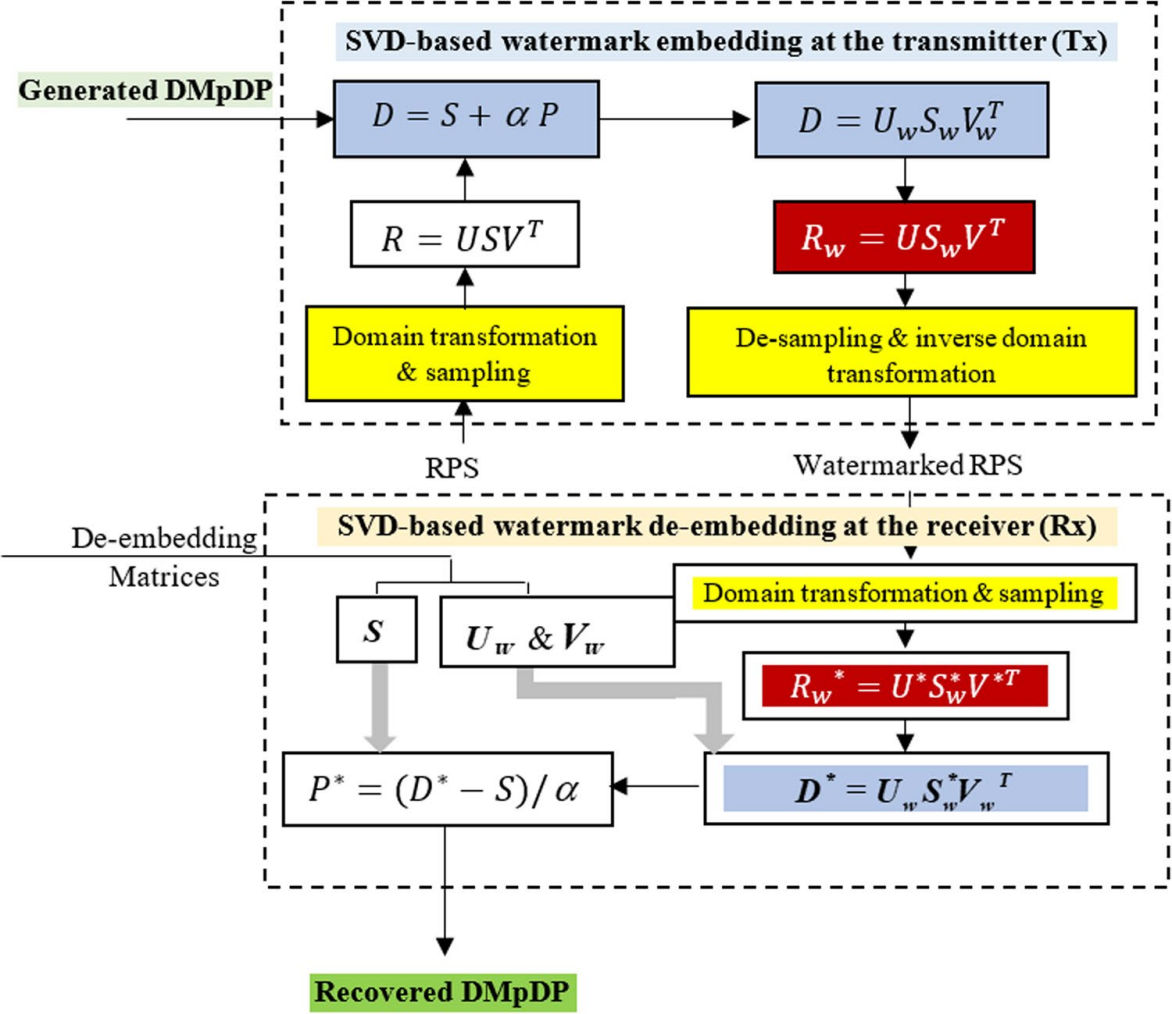
SVD-based watermark embedding and de-embedding process

Figure 4 demonstrates the SVD-based watermark embedding and de-embedding process.

### Guided SAF teledermoscopy system using the Diagnostic Multiple-patient DermoFeature Profile

In the proposed DMpDP-based guided SAF teledermoscopy system, a CAD (computer-aided diagnosis) system is employed based on the DMpDPs. Several operation modes can be applied using the proposed system, namely the patient-physician mode, the patient-system mode, the physician-physician mode, and the physician-system mode.

In the patient-physican mode, the patient captures one image or a sequence of images for the suspicious lesion. Then, these images are transformed into the proposed DMpDP and inserted as a watermark in a recorded patient information record. The watermarked RPS is transmitted over the teledermoscopy channel to the physician, who exploits both the retrieved DMpDP and RPS in performing this diagnosis. The proposed system provides the classification results as guidance to the physician for the final decision. Hence, in the patient-system mode, the user (i.e., the patient) receives the diagnosis based on the proposed automated classification decision of the pre-trained classifier using the retrieved DMpDP only.

**Fig. 5:**
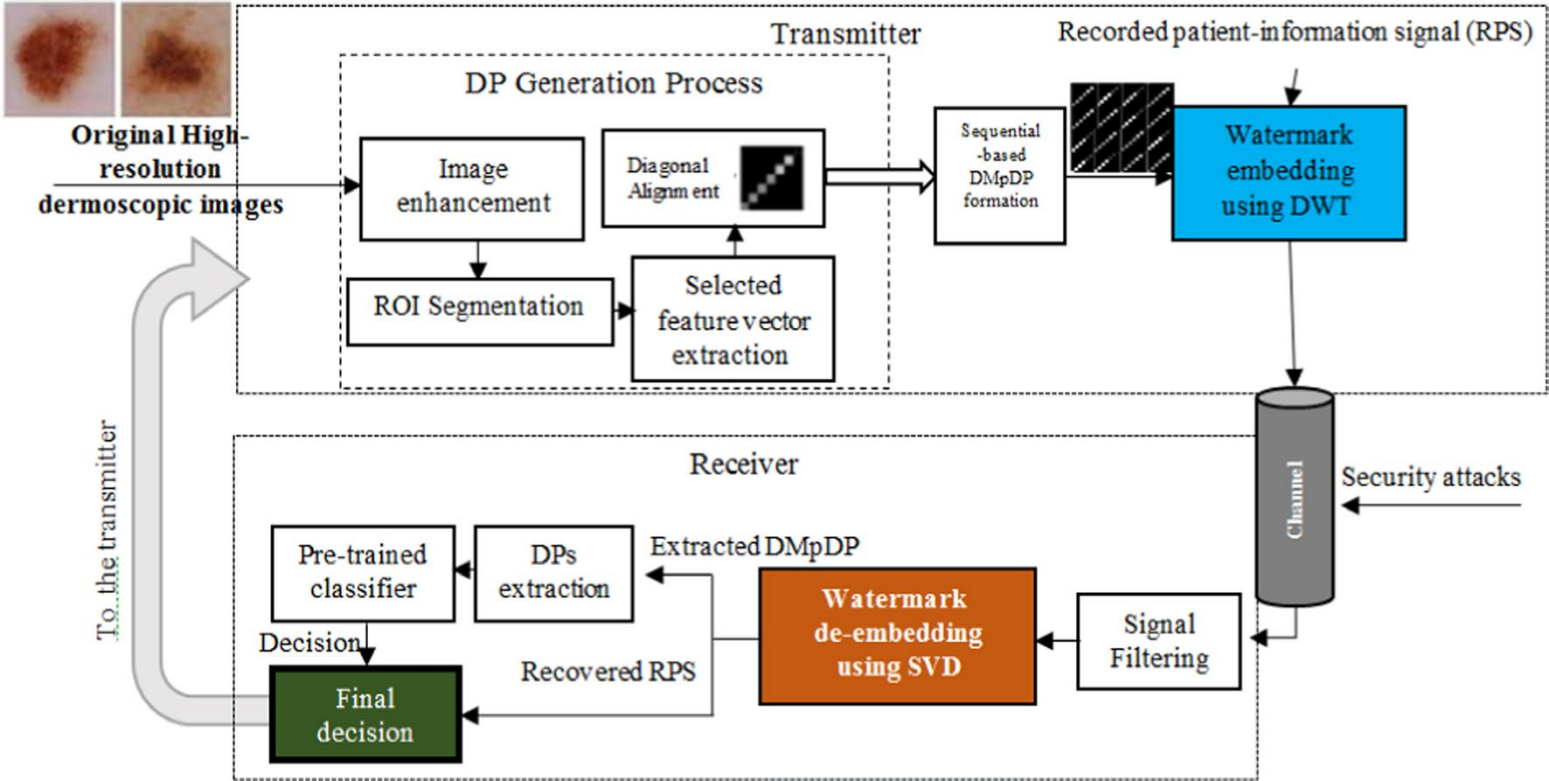
The proposed DMpDP-based guided SAF teledermoscopy system

In the physician-physician mode, also known as the consultation mode, the DMpDPs representing the lesions of multiple patients or a single patient over some time are transmitted to a remote physician or medical facility for a second opinion. In this case, the DMpDP is embedded in an RPS which carries more refined medical information, such as the patient(s) demographics, the patient(s) history, symptoms/complaints, and the history of the patient(s) tumors. Therefore, the remote physician can provide the diagnosis based on his/ her expertise in addition to the proposed automated classification decision, which acts as a decision support system to guide the physician. Nevertheless, in the physician-system mode, the physician receives the automated diagnosis for the patient(s) from the pre-trained classifier based on the DMpDP only. Figure 5 outlines the workflow of the proposed DMpDP-based guided SAF teledermoscopy system.

The transmitter is responsible for the formation and embedding of the DMpDP in the RPS. As it is shown by the dotted box in Fig. 5, each fed high-resolution original image is initially processed to obtain its corresponding DP. Image enhancement, ROI segmentation using wavelet transform-based thresholding, and extraction of selected features are performed sequentially for each original image. Then, the feature vector is aligned diagonally forming the DP for a single image. As the proposed system targets the collation of a group of DPs for providing a wide-scale service, the generated DPs are organized into the sequential-based DMpDP form. The DMpDP is embedded in the recorded patient-information signal using the DWT transform-based SVD watermarking technique before transmission over the teledermoscopy channel. The received data is stored in a database which is accessed by the receiver’s workstation for checking the upcoming requests and responding to them, in addition to the experimentation and surveying purposes. Accordingly, the received watermarked signal at the receiver is processed at the filtering stage followed by extracting the watermark (i.e., DMpDP) and retrieving the RPS using the SVD watermark extraction technique. The DPs are extracted and fed to the pre-trained second-order polynomial support vector machine classifier to produce the automated decision. In the patient-physician and physician-physician modes, the medical information in the RPS is employed to provide the final decision to the user.

### Evaluation metrics

The performance of the horizontally aligned DMpDP, the diagonal-based DMpDP, and the sequential-based DMpDP were studied in respect of the recorded signal quality metrics of the SVD-watermarked RPS for different domains: time, DWT, DST, and DCT using the log-likelihood ratio (LLR) which is used in the statistical hypothesis testing to measure the strength of evidence for the original speech signal compared to the watermarked speech signal

14 $${{L}}{{L}}{{R}}=\left|{{l}}{{o}}{{g}}\left(\frac{{{{l}}}_{{{s}}}{{{R}}}_{{{y}}}{{{l}}}_{{{s}}}}{{{{l}}}_{{{y}}}{{{R}}}_{{{y}}}{{{l}}}_{{{y}}}}\right)\right|$$

where ${l}_{s}$ and ${l}_{y}$ are the coefficient vectors for the LPC for the original and the watermarked recorded signals, respectively. Ry matrix is the autocorrelation of the watermarked speech signal. Also, the spectral distortion (SD) quantifies the difference between the original spectral content of a speech signal and the watermarked spectral content.

15 $${{S}}{{D}}=\frac{1}{{{N}}}\sum_{0}^{{{N}}-1}\sum_{{{k}}={{{n}}}_{{{s}}}{{m}}}^{{{{n}}}_{{{s}}}{{m}}+{{{n}}}_{{{s}}}-1}\left|{{S}}\left({{k}}\right)-{{Y}}({{k}})\right|$$

where $S(k)$ and $Y(k)$ are the original spectra and wat $R$ ermarked recorded signal spectra in dB. The signal-to-noise ratio (SNR) measures the ratio of the power or energy of the original speech signal to the mean square error between the original and the watermarked speech signal, and the RPS correlation coefficient ${C}_{s}$ evaluates the correlation between the original and watermarked speech signal. Also, the integrity of the extracted watermark (i.e., DMpDP) using the SVD at the receiver end was evaluated using the watermark correlation coefficient ${C}_{w}$.

Moreover, the presented system was evaluated with the existence of an additive white Gaussian noise (AWGN) attack for the teledermoscopy channel. The AWGN attack involves injecting random noise into the watermarked signal to degrade the quality of the watermark or render it undetectable. However, an attacker may try to undermine the effectiveness of the watermark by introducing AWGN to the watermarked signal. AWGN is a form of noise that conforms to a Gaussian distribution, exhibiting random amplitude and a consistent power spectral density across all frequencies. Since the noise was added to the original watermarked signal, it is considered additive. The attacker’s goal in adding AWGN is to corrupt the embedded watermark and make its accurate extraction more difficult. The attacker introduces random noise to the watermarked signal, with the noise level or intensity typically specified using SNR, often measured in decibels (dB).

## Results

**Fig. 6:**
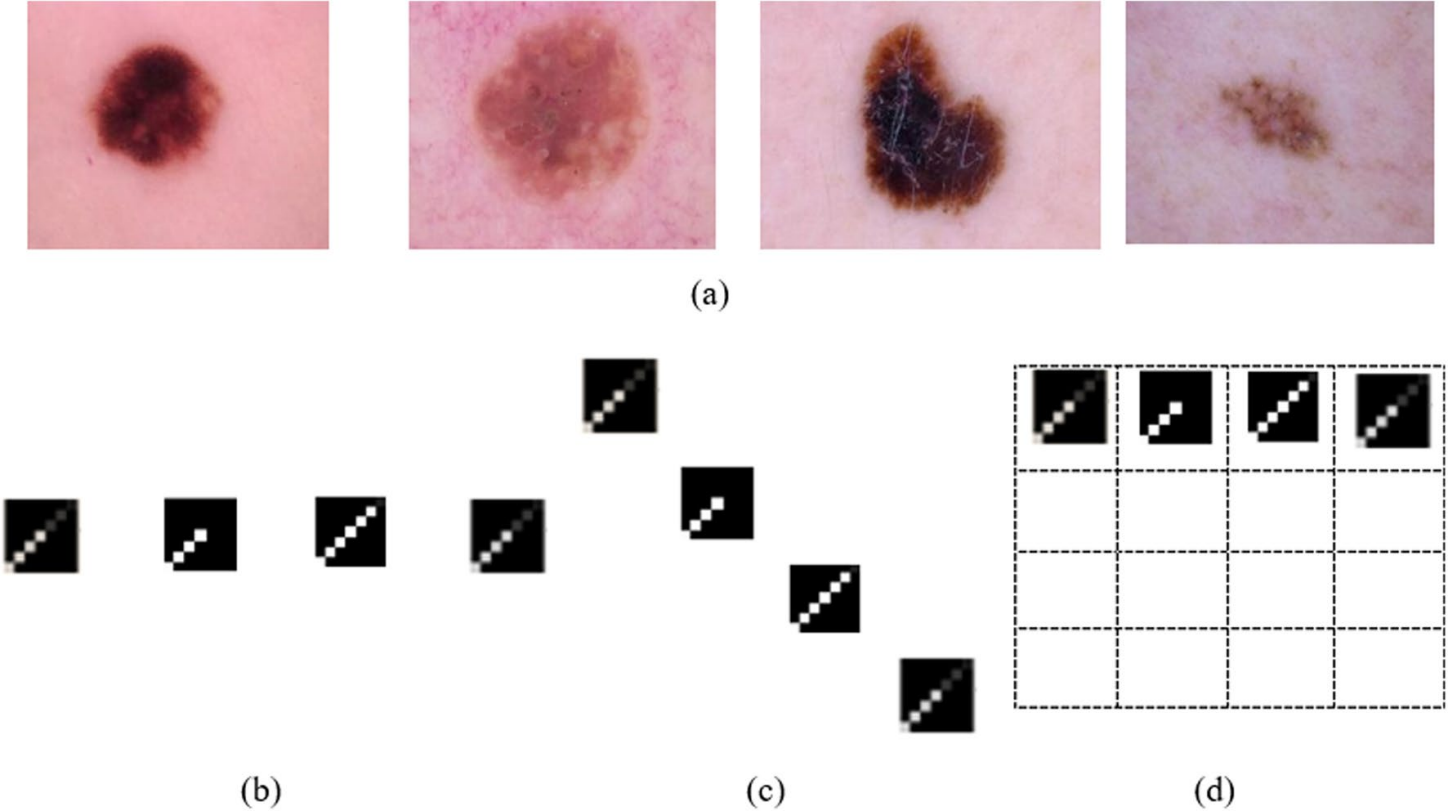
Sample dermoscopy images and their corresponding DMpDPs, where dermoscopy images (**a**); horizontally aligned DMpDPs (**b**); diagonal-based DMpDPs (**c**); and sequential-based DMpDPs (**d**)

The CAD system was trained using five-fold cross-validation. The feature selection process ranked the extracted WIDF and MOF feature sets to pick the most significant features. The experiments established the high performance of the second-order polynomial SVM using the selected features resulting in high classification accuracy. The SVM model used the sequential minimal optimization solver and had a box constraint value of 1, a kernel scale of 1, and a kernel offset of 0. The performance of the different formations of DMpDPs was studied in respect of the recorded signal quality metrics of the watermarked RPS for different domains: time, DWT, DST, and DCT using the log-likelihood ratio (LLR). Moreover, the presented system was evaluated with the existence of the AWGN attack for the teledermoscopy channel. Figure 6 displays sample high-resolution dermoscopy images from our dataset in addition to sample DMpDPs in horizontally aligned, diagonal-based, and sequential-based forms. Figure 6d represents the sequential-based form for the four sample images, however, with a larger number of images, the remaining rows of the DMpDPs shall hold the DPs for the corresponding images.

We aimed to discover the best of the DMpDP, the best transform domain for the SVD watermarking in terms of RPS quality and extracted watermark integrity, the optimal number of collated DPs within the DMpDP, and the effect of the *α* and *β* values on the system performance with the existence of AWGN attack where α is the weight of embedding the watermark on the speech signal and β is the weight multiplied by the RPS signal to amplify it before the embedded process. Four scenarios were implemented to investigate them.

### Scenario (0): Study the effect of spacing between the DPs of the horizontally aligned DMpDP using ***α*** = 0.01 and *β* = 1 on RPS quality

For this scenario, the dataset images were divided into two subsets: the first was used to generate the first (i.e. leftmost) DP, while the second was used to generate the second (i.e. rightmost) DP to present a two-DP horizontally aligned DMpDPs (Fig. 1). The SVD transform-based watermarking of the proposed horizontally aligned DP involves two main factors: the selected transform domain of the watermarking technique and the effect of the spacing (i.e., number of samples) between the two embedded DPs in the RPS on the RPS quality. Hence, the different transform domains were investigated in addition to the effect of different spacing values on the recorded signal quality. Spacing values of 0, 25, 50, and 75 samples were considered. In this scenario, *α* = 0.01 is used to reduce the watermark weight, while preserving the weight of the RPS by assigning *β* = 1.

**Fig. 7:**
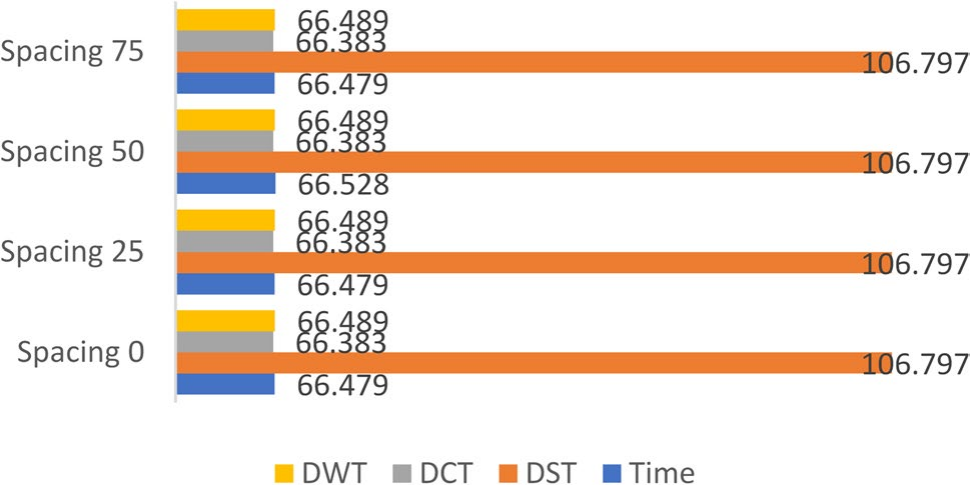
Average signal-to-noise ratio of the watermarked recorded signal for horizontally aligned DMPDP representation for different spacing

**Fig. 8:**
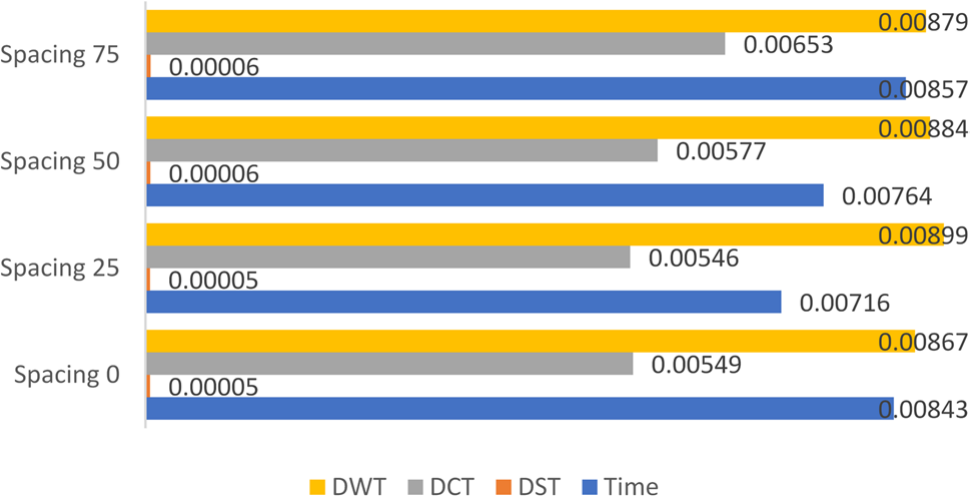
Average spectral distortion of the watermarked recorded signal for horizontally aligned DMPDP representation for different spacing

The main purpose of scenarios (0) and (1) is to assess the RPS quality metrics for the lowest watermark weight, as an initial validation for the proposed concept. Figures 6, 7, and 8 demonstrate the average values for SNR, SD, and LLR of the watermarked RPSs used over all the dataset using the horizontally aligned DMpDP for different embedding transform domains and spacing values.

Figure 7 demonstrates that the highest average SNR value 106.8 was obtained using DST-based SVD watermarking, while the DCT, DWT, and time domains resulted in nearly similar average SNR values of approximately 66.4.

Figure 8 shows that the DST-based SVD achieved the least average SD of nearly 5×10^–5^. Higher SD values were obtained using the time, DCT, and DWT domains, such that the least SD value obtained using the DCT-based SVD was 0.0055, followed by the time-based SVD having a minimum SD 0.0072, while the DWT-based SVD achieved a minimum SD 0.0086.

**Fig. 9:**
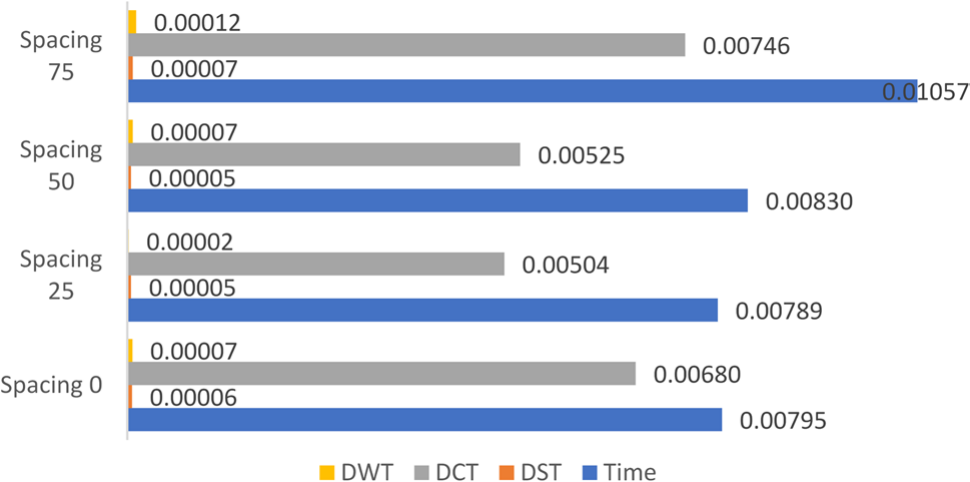
Average log-likelihood ratio of the watermarked recorded signal for horizontally aligned DMPDP representation for different spacing

Figure 9 shows that the least average values of the LLRs were obtained using the DWT and the DST domains, having a minimum 1.77 × 10^–5^ and 5 × 10^–5^, respectively.

From Figs. 7, 8, and 9, it can be concluded that the DST-based SVD watermarking achieves the most optimal recorded signal quality metrics. In terms of spacing, Figs. 8 and 9 indicate the least SD values using the DST-based SVD watermarking for spacing 25, followed by spacing 0, 50, and 75. The least LLR values using the DST-based SVD watermarking were obtained for spacing 50, 25, 0, and 75. However, the given spacing values had a minor effect on the SNR values as displayed in Fig. 7. Yet, the recorded signal correlation coefficient ${C}_{s}=1$ in all of the previous cases due to the low value of *α*. By considering these findings, it is concluded that a spacing value 25 represents a fair compromise between the RPS quality metrics using the DST-based SVD watermarking.

### Scenario (1): Study the effect of the number of DPs in the horizontally aligned DMpDP using spacing 25 samples, $\alpha =0.01$ and *β* = 1 on RPS quality

Scenario (1) aims to investigate the effect of the number of DPs within the proposed horizontally aligned DMpDP on the recorded signal quality metrics to conclude the maximum number of DPs that can be collated within the horizontally aligned DMpDP. In scenario (0), two DPs were collated to form a single horizontally aligned DMpDP. However, in scenario (1), the effect of having 12, 48, 96, and 248 DPs per DMpDP on the RPS quality for spacing 25 was investigated and applied for all dataset images that transmit in multiple recorded signals to measure the mean performance of the recorded signal quality. For example, in the case of 12 DPs, each class contributes with three DPs, e.g., three patients, and was embedded in one recorded signal after that this process was repeated for all dataset images to measure the mean quality. Table 1 displays the average values for SNR, LLR, and SD of the watermarked RPS with the proposed horizontally aligned DMpDP using different transform domains for SVD watermarking for different numbers of DPs.

**Table 1:** Average quality metric of the recorded signal of the watermarked RPS with the proposed horizontally aligned DMpDP for different numbers of DPs

	SNR	LLR	SD
Time-domain-based SVD watermarking			
12 DPs	58.6392	0.020798	0.022849
48 DPs	52.57345	0.096581	0.045957
96 DPs	49.55431	0.218081	0.067456
248 DPs	45.43792	0.371632	0.107917
DST-based SVD watermarking			
12 DPs	98.99601	0.000105	0.000206
48 DPs	92.97239	0.000208	0.000438
96 DPs	89.96139	0.000171	0.000639
248 DPs	85.84434	0.000163	0.001016
DCT-based SVD watermarking			
12 DPs	58.59657	0.011649	0.022143
48 DPs	52.56862	0.022978	0.046548
96 DPs	49.55253	0.021679	0.06698
248 DPs	45.43212	0.032149	0.105983
DWT-based SVD watermarking			
12 DPs	58.61205	0.000699	0.023538
48 DPs	52.56564	0.007758	0.04794
96 DPs	49.552	0.010089	0.067534
248 DPs	45.43438	0.043101	0.107174

Table 1 reveals that the average metrics of the recorded signal quality metrics decrease with the increase in the number of DPs. For example, using the DST-based SVD watermarking, the average SNR achieved 98.99, 92.97, 89.96, and 85.84 with 12, 48, 96, and 248 DPs (patients), respectively. A trend of increasing the average SD and the average LLR was observed with the increase in the number of DPs. Therefore, similarly to the conclusion in scenario (0), the DST-based SVD watermarking achieved the best recovered recorded signal quality compared to the other domains. It is worth noting that the average correlation coefficient ${C}_{s}$ of the recorded signal had a value of nearly 1 in all scenarios due to low *α*.

From this scenario (1), it can be concluded that for the proposed horizontally aligned DMpDP, the recorded signal quality is severely degraded by the increase in the number of DPs, which is mostly reflected in the average SD values.

### Scenario (2): Study the effect of the number of DPs in the diagonal-based and the sequential-based DMpDPs using *α* = 0.01 and *β* = 1 without channel noise on RPS quality

Based on the limitation on the number of DPs within the horizontally aligned DMpDP, the effect of the number of DPs within the diagonal-based DMpDP and the sequential-based DMpDP were also investigated. Hence, in this experiment, *α* = 0.01, and *β* = 1.

Using diagonal-based DMpDP, the single DP image has size 7 × 7. while the used recorded signal includes 22,000 samples. Hence, for the diagonal-based DMpDP, the maximum size of the DMpDP is the square root of the number of recorded signal samples, which is 148. The maximum number of DPs within the diagonal-based DMpDP according to the given recorded signal is 21 DPs (i.e., 148 divided by 7). Therefore, the cases of having 12 DPs and 20 DPs were studied, as they correspond in having 3 and 4 DPs per class, respectively. Table 2 displays the average values of SNR, LLR, and SD of the watermarked RPS over all the dataset images with the proposed diagonal-based DMpDP using different transform domains for SVD watermarking for different numbers of DPs.

**Table 2:** Recorded signal quality metrics of the watermarked RPS with the proposed diagonal-based DMpDP for different numbers of DPs

	SNR	LLR	SD
Time-domain-based SVD watermarking			
12 DPs	58.61655	0.275378	0.023611
20 DPs	56.4011	0.041035	0.030124
DST-based SVD watermarking			
12 DPs	98.99601	1.64E-05	0.000224
20 DPs	96.77794	2.9E-05	0.00029
DCT-based SVD watermarking			
12 DPs	58.59556	0.001906	0.023387
20 DPs	56.37572	0.001892	0.030284
DWT-based SVD watermarking			
12 DPs	58.58598	0.001273	0.023511
20 DPs	56.38102	0.027946	0.030749

Table 2 demonstrates that the DST is the best transform domain for the SVD watermarking in terms of recorded signal quality, as it achieves average SNRs from 96.77 to 98.99, while the DCT, DWT, and the time domains achieved average SNRs from 56.37 to 58.62. Also, the average LLR values of the DST domain were from $1.6\times {10}^{-5}$ to $2.9\times {10}^{-5}$, while the other domains achieved average LLR values from 0.0018 to 0.275. The least average SD values were also observed for the DST domain, as the average SD values were nearly 0.0002, while the other domains’ SD values were from 0.023 to 0.030. It was evident that better metrics were obtained in the case of 12 DPs compared to 20 DPs for all transform domains. Hence, it can be established that the proposed diagonal-based DMpDP can be used with a few number of patients, where the top performance was achieved using less number of DPs.

For the sequential-based DMpDP, to obtain the proposed matrix fairly carrying the DPs of all classes, each row of the matrix was assigned the DPs of a certain class, which results in having four rows. Hence, the maximum size of the DMpDP is equal to the number of samples in the recorded signal divided by the number of classes multiplied by the number of samples per DP column (i.e., 4 × 7), which results in a maximum of 785 samples per the DMpDP column, which is equivalent to 112 DPs. Therefore, cases of having multiples of four DPs were studied, such as 12, 48, 96, and 248 DPs. Table 3 displays the average values of SNR, LLR, and SD of the watermarked RPS with the proposed sequential-based DMpDP using different transform domains for SVD watermarking for different numbers of DPs.

**Table 3:** Average quality metrics of recorded signal of the watermarked RPS with the proposed sequential-based DMpDP for different numbers of DPs

	SNR	LLR	SD
Time-domain-based SVD watermarking			
12 DPs	64.40107	0.012814	0.011797
48 DPs	62.53639	0.095788	0.014736
96 DPs	62.4464	0.014575	0.015004
248 DPs	62.03616	0.002456	0.015921
DST-based SVD watermarking			
12 DPs	105.3324	6.44E-05	0.000109
48 DPs	103.7684	1.16E-05	0.000129
96 DPs	103.8875	7.38E-06	0.000126
248 DPs	103.4882	4.2E-07	0.000133
DCT-based SVD watermarking			
12 DPs	63.57467	0.009081	0.013146
48 DPs	56.80779	0.001129	0.028521
96 DPs	53.72022	0.000736	0.039988
248 DPs	49.38283	4.91E-05	0.06782
DWT-based SVD watermarking			
12 DPs	64.57648	0.001041	0.011715
48 DPs	63.14224	0.000696	0.013809
96 DPs	63.27903	0.000591	0.013693
248 DPs	62.71788	0.000332	0.014469

Table 3 demonstrates that the DST is the best transform domain with the SVD watermarking in terms of preserving the recorded signal quality, as it achieves average SNRs from 103.48 to 105.33, while the DCT, DWT, and the time domains achieved average SNRs from 49.38 to 64.58. Also, the average LLR values of the DST domain were from $4.2\times {10}^{-7}$ to $6.44\times {10}^{-5}$ On the contrary, the other domains achieved average LLR values from $4.91\times {10}^{-5}$ to $0.0957$. The least average SD values were also observed with the DST domain, as the average SD values were nearly 0.0001, while the other domains’ SD values were from 0.000109 to 0.06782. It was evident that the recorded signal quality metrics were relatively constant with the increase in the number of DPs, as reflected by the average SNR and average LLR values.

From the above results and by comparing the average quality metrics of the recorded signal quality metrics for the proposed three DMpDP forms, it was concluded that the sequential-based form provided the best results with the increase in the number of DPs followed by the diagonal-based and the horizontally aligned forms. Also, it was concluded that applying the DST domain during the embedding process provided the best metrics of the recorded signal quality.

### Scenario (3): Study the effect of ***α*** and *β* on the classification accuracy of the diagonal-based and the sequential-based DMpDPs with the existence of AWGN attack

In this scenario, the classification performance of the presented system was assessed for *α* = 0.01, 0.1, 0.5, and 1, in addition to *β* = 2, 10, 100, 200, 300, 400, 500, and 1000. The main objective was to evaluate the effect of these values on both the diagonal-based and the sequential-based DMpDPs using all the dataset images to compare the effect of the proposed *β* factor to *α*. Also, scenario (3) aimed to study the outcome of selecting the dissimilar transformations on the watermark integrity and the classification accuracy. This scenario proposed the existence of an AWGN attack on the teledermoscopy channel considering SNRs − 5, 0, and + 5 dBs.

For diagonal-based DMpDP using time-domain SVD watermarking, as shown by Table 4, the highest accuracies were obtained using the DWT-based SVD watermarking with *α* = 1 and *β* = 10. For 12 DPs, the classification accuracies were 33.25%, 36.72%, and 37.41% at − 5, 0, and + 5 dB, respectively. These values have reached 29.39%, 31.58%, and 35.96% for − 5, 0, and + 5 dB, respectively, for 20 DPs. From Table 4, we conclude that the DWT domain resulted in the highest classification accuracy compared to the other embedding domains and, hence, the highest watermark correlation coefficient. The proposed recorded signal weight *β* is more impactful in improving classification performance compared to the watermark weight *α*.

**Table 4:** $\alpha$ and $\beta$ values resulting in the highest classification accuracies at the different embedding domains for diagonal-based DMpDP under AWGN attack.

	− 5 dB	0 dB	+ 5 dB	*α*	*β*
Time-domain-based SVD watermarking					
12 DPs	32.63%	36.71%	42.44%	1	10
20 DPs	29.47%	33.42%	35.79%		
DST-based SVD watermarking					
12 DPs	26.39%	26.74%	26.82%	1	10
20 DPs	26.67%	26.93%	26.14%	1	100
DCT-based SVD watermarking					
12 DPs	14.23%	13.63%	13.98%	1	1000
20 DPs	10.79%	11.14%	10.52%		
DWT-based SVD watermarking					
12 DPs	33.25%	36.72%	37.41%	1	10
20 DPs	29.39%	31.58%	35.96%		

For sequential-based DMpDP, as shown in Table 5, using time-domain SVD watermarking, the highest accuracies were obtained using *α* = 1 and *β* = 1000. For 4 DPs, the classification accuracies were 76.91% at − 5 dB, 78.65% at 0 dB, and 79.34% at + 5 dB. These values increased in the case of transmitting 12 DPs to 79.17%, 80.90%, and 82.12% for − 5, 0, and + 5 dB, respectively. Likewise, with 24 DPs, the classification accuracies reached 87.33%, 87.93%, and 88.37% for the same SNR ratios, respectively. The classification accuracies have further increased with the increase in the number of DPs reaching 94.01%, 94.18%, and 94.09%, with 48 DPs, while achieving 96.7%, 97.14%, and 97.48%, with 96 DPs, and 98.69%, 98.79%, and 98.99%, with 248 DPs.

**Table 5:** $\alpha$ and $\beta$ values resulting in the highest classification accuracies at the different embedding domains for sequential-based DMpDP under AWGN attack

	− 5 dB	0 dB	+ 5 dB	*α*	*β*
Time-domain-based SVD watermarking					
4 DPs	76.91%	78.65%	79.34%	1	1000
12 DPs	79.17%	80.90%	82.12%		
24 DPs	87.33%	87.93%	88.37%		
48 DPs	94.01%	94.18%	94.09%		
96 DPs	96.7%	97.14%	97.48%		
248 DPs	98.69%	98.79%	98.99%		
DST-based SVD watermarking					
4 DPs	76.48%	76.21%	76.13%	1	1000
12 DPs	75.78%	79.34%	79.86%		
24 DPs	81.59%	84.80%	86.55%		
48 DPs	93.92%	93.75%	93.66%		
96 DPs	96.78%	96.96%	97.14%		
248 DPs	98.59%	98.99%	98.69%		
DCT-based SVD watermarking					
4 DPs	75.61%	77.08%	76.82%	1	1000
12 DPs	71.27%	74.31%	79.07%		
24 DPs	61.72%	63.63%	69.27%		
48 DPs	57.29%	58.68%	65.36%		
96 DPs	54.77%	56.51%	63.46%		
248 DPs	52.52%	54.23%	60.99%		
DWT-based SVD watermarking					
4 DPs	76.74%	77.08%	77.43%	1	1000
12 DPs	81.59%	81.16%	81.07%		
24 DPs	87.32%	88.02%	88.72%		
48 DPs	93.66%	94.27%	93.58%		
96 DPs	97.05%	97.48%	97.14%		
248 DPs	98.79%	98.79%	98.99%		

Using DWT-domain SVD watermarking, the highest accuracies are obtained using *α* = 1 and *β* = 1000. For 4 DPs, the classification accuracies are 76.74% at − 5 dB, 77.08% at 0 dB, and 77.43% at + 5 dB. These values increased with transmitting 12 DPs to obtain 81.59%, 81.16%, and 81.07% at − 5, 0, and + 5 dB, respectively. In addition, for 24 DPs, the classification accuracies reached 87.32, 88.02, and 88.72 for the same SNR ratios, respectively. The classification accuracies have further increased with the increase in the number of DPs reaching 93.66, 94.27, and 93.58 for 48 DPs while achieving 97.05, 97.48, and 97.14 for 96 DPs, and 98.79, 98.79, and 98.99 for 248 DPs. However, less results were obtained using the DCT-based and DST-based watermarking.

### Scenario (4): Study the effect of *β* and filtering techniques on the classification accuracy of the sequential-based DMpDPs with the existence of AWGN attack

In scenario (4), only the sequential-based form was considered, and the reduction of *β* was investigated to decrease the system’s complexity. Also, the effect of using a filtering stage, such as Weiner, adaptive Weiner, spectral subtraction, and wavelet denoising techniques, was studied on all the dataset images.

**Fig. 10:**
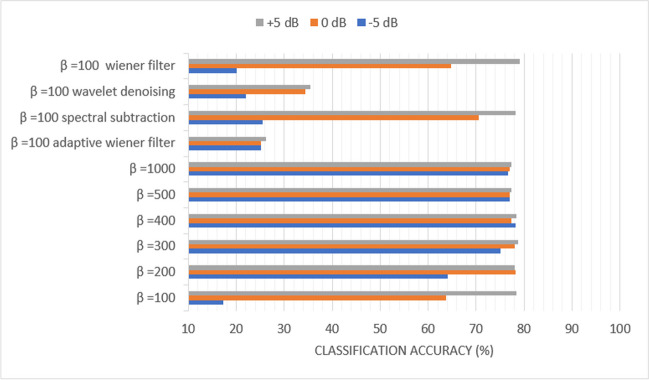
Classification accuracy of the 4 DP-sequential-based DMpDPs for different *β* values and filtering techniques

**Fig. 11:**
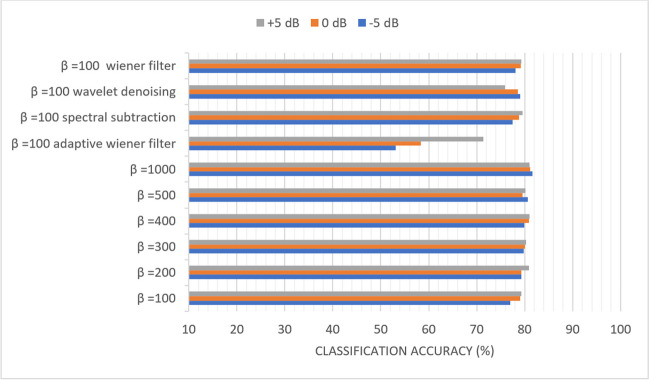
Classification accuracy of the 12 DP-sequential-based DMpDPs for different *β* values and filtering techniques

**Fig. 12:**
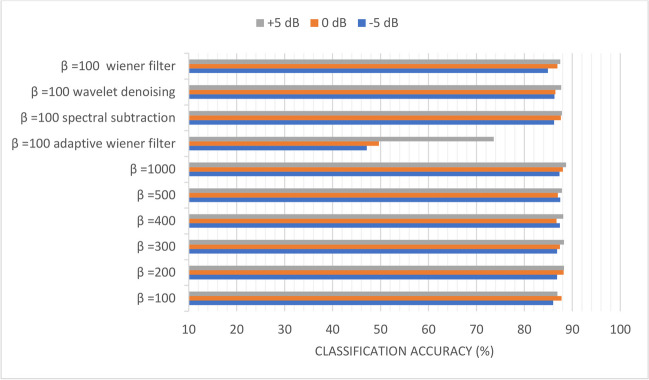
Classification accuracy of the 24 DP-sequential-based DMpDPs for different *β* values and filtering techniques

**Fig. 13:**
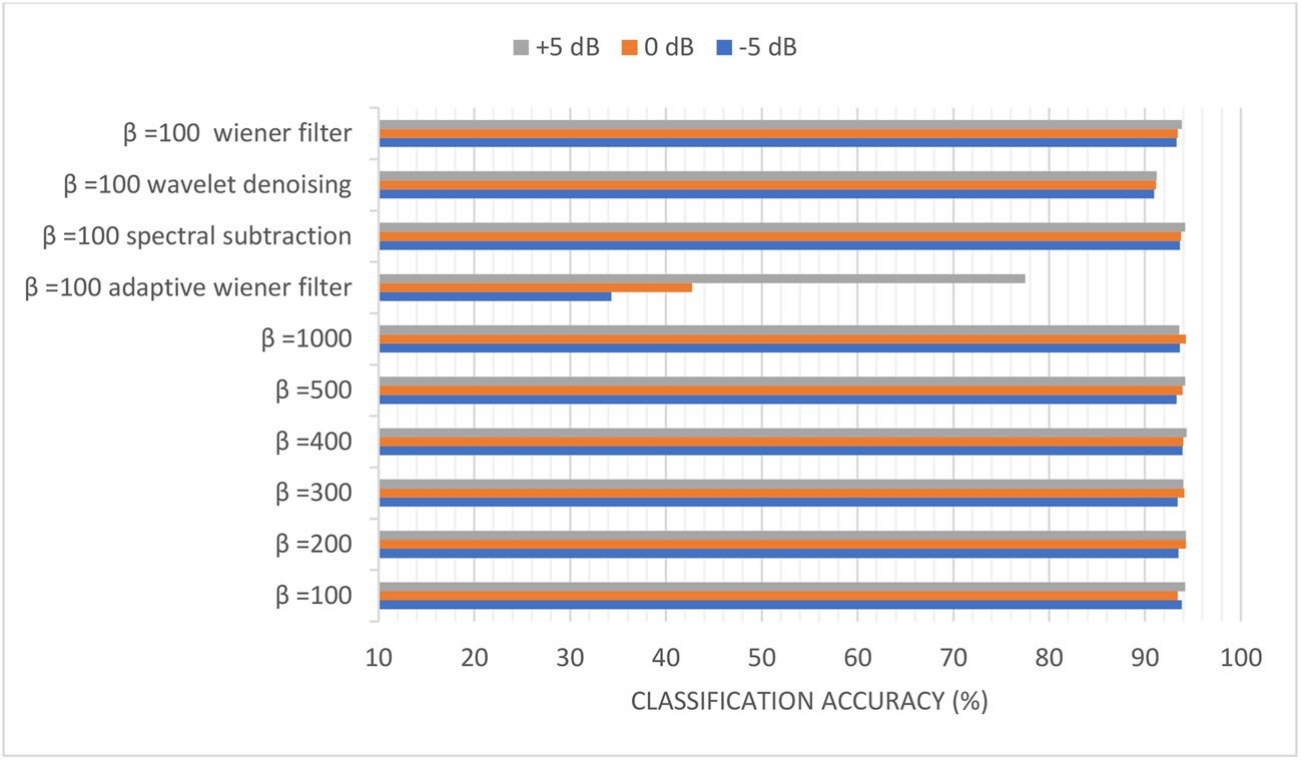
Classification accuracy of the 48 DP-sequential-based DMpDPs for different *β* values and filtering techniques

**Fig. 14:**
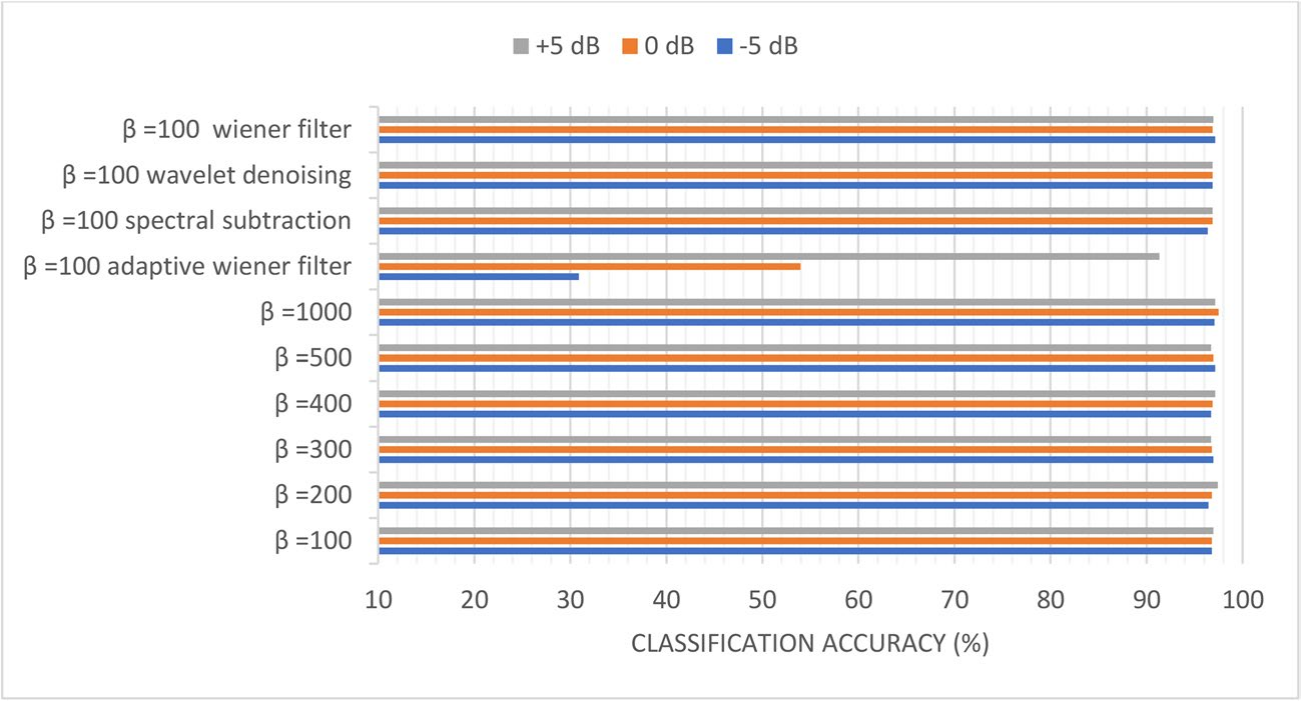
Classification accuracy of the 96 DP-sequential-based DMpDPs for different *β* values and filtering techniques

**Fig. 15:**
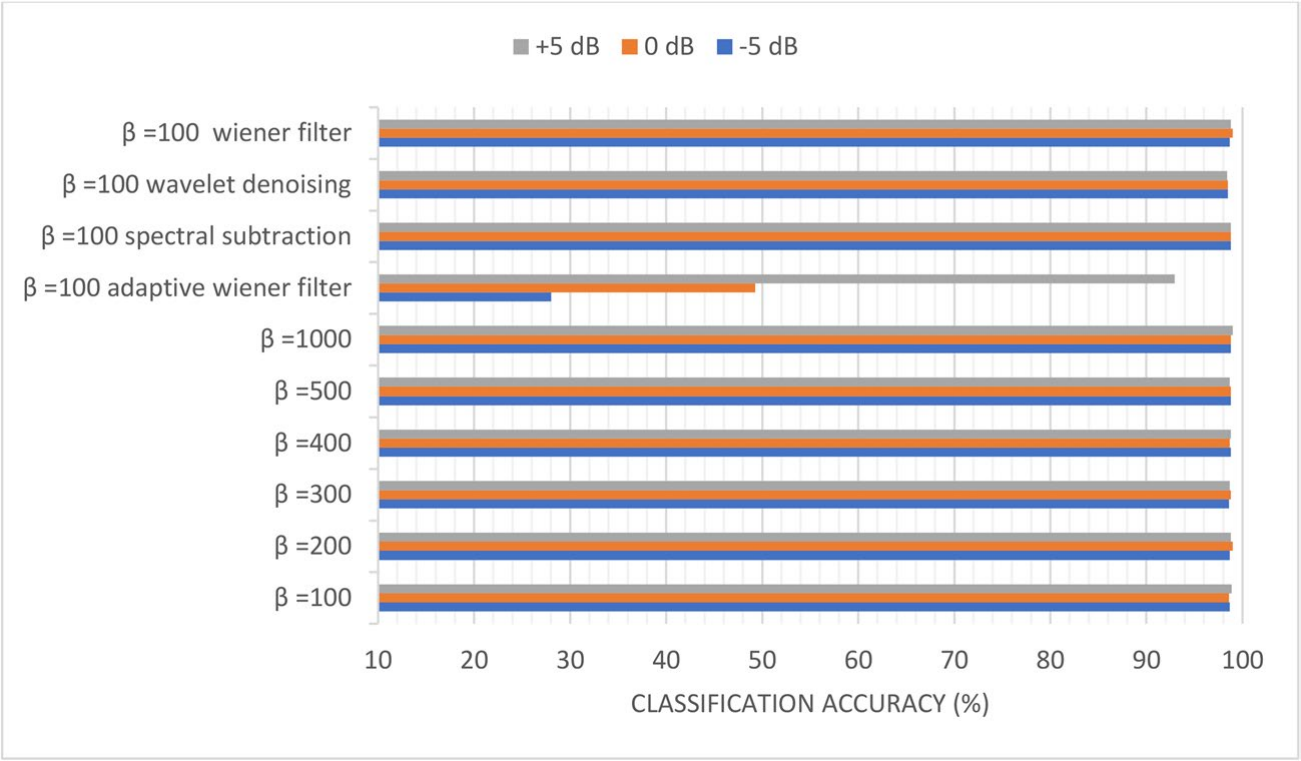
Classification accuracy of the 248 DP-sequential-based DMpDPs for different *β* values and filtering techniques

Figures 10, 11, 12, 13, 14, and 15 display the classification accuracy of the sequential-based DMpDPs having 4, 12, 24, 48, 96, and 248 DPs, respectively, for *β* 100, 200, 300, 400, 500, and 1000, and in the presence of filtering techniques for *β* = 100. *α* = 1, and only the DWT domain was considered as it realized the best classification performance from scenario (3). Accordingly, Figs. 10, 11, and 12 illustrate the classification accuracy of transmitting different numbers of DPs in the form of sequential-based DMpDPs for different *β* values and using different filtering techniques.

Figures 10, 11, and 12 prove that increasing the values of *β* leads to higher performance compared to the use of the given filtering techniques in the case of 4, 12, and 24 DPs. For example, in Fig. 10, at − 5 dB, the accuracy increased from 17.27% for *β* = 100 to 76.7% at *β* = 1000, while the highest accuracy was obtained with filtering at 25.4%, using spectral subtraction. Similarly, in Fig. 11, the accuracy has increased from 76.99% for *β* = 100 to 81.59% for *β* = 1000, while the highest accuracy obtained with filtering was 79.07%, using wavelet denoising.

In Fig. 12, the accuracy has increased from 86.02% for *β* = 100 to 87.33% for *β* = 1000, while the highest accuracy obtained with filtering was 86.28%, using wavelet denoising. Figures 13, 14, and 15 report the effect of increasing the number of the transmitted DPs on the classification accuracy in the case of the sequential-based DMpDPs using different *β* values and filtering techniques.

Figure 15 illustrates the mean classification accuracy over all the datasets after being transmitted and received at the receiver. Table 6 demonstrates in detail the classification accuracy for four received 248 images for 248 DP-sequential-based DMpDPs. At each embedding process for 248 DP-sequential-based DMpDPs, 248 DP-sequential-based DMpDPs consist of a combined 62 DP from each four classes and are embedded in the recorded speech signal as 248 DP-sequential-based DMpDPs. At the receiver, the 248 DP-sequential-based DMpDPs were extracted from the recorded speech signal and measured the classification accuracy over the received 248 DP.

**Table 6:** The mean classification accuracy in details for transmitting four of the 248 DP-sequential-based DMpDPs for different *β* values and filtering techniques

Filter	Transmitted images	− 5 dB	0 dB	+ 5 dB	*α*	*β*
Without filter	1st 248 DP	98.39	98.39	98.39	1	100
	2nd 248 DP	98.79	98.39	98.79		
	3rd 248 DP	98.79	99.19	99.19		
	4th 248 DP	98.79	98.39	99.19		
Without filter	1st 248 DP	98.39	98.39	98.39	1	200
	2nd 248 DP	98.39	98.79	98.79		
	3rd 248 DP	99.19	99.19	98.79		
	4th 248 DP	98.39	99.19	98.79		
Without filter	1st 248 DP	98.39	98.39	98.39	1	300
	2nd 248 DP	98.79	98.79	98.79		
	3rd 248 DP	99.19	98.79	98.79		
	4th 248 DP	99.19	98.79	98.39		
Without filter	1st 248 DP	98.39	98.39	98.39	1	400
	2nd 248 DP	98.79	98.79	99.19		
	3rd 248 DP	98.79	98.79	98.79		
	4th 248 DP	98.79	98.39	98.79		
Without filter	1st 248 DP	98.39	98.39	98.79	1	500
	2nd 248 DP	98.79	99.19	99.19		
	3rd 248 DP	98.79	98.79	98.79		
	4th 248 DP	98.39	98.79	98.39		
Without filter	1st 248 DP	98.39	98.79	99.19	1	1000
	2nd 248 DP	99.19	99.19	98.79		
	3rd 248 DP	98.79	98.79	98.79		
	4th 248 DP	98.79	98.39	98.39		
Adaptive wiener filter	1st 248 DP	29.03	48.79	93.14	1	100
	2nd 248 DP	27.82	50.01	91.94		
	3rd 248 DP	27.02	47.98	93.95		
	4th 248 DP	28.23	50.41	92.74		
Spectral subtraction filter	1st 248 DP	98.79	98.79	98.79	1	100
	2nd 248 DP	98.79	98.79	98.79		
	3rd 248 DP	98.79	98.79	98.79		
	4th 248 DP	98.79	98.79	98.79		
Wavelet denoising	1st 248 DP	98.39	98.39	98.39	1	100
	2nd 248 DP	98.39	98.39	98.39		
	3rd 248 DP	98.39	98.79	98.39		
	4th 248 DP	98.79	98.39	98.39		
Wiener filter	1st 248 DP	98.79	99.59	99.19	1	100
	2nd 248 DP	98.39	98.39	98.39		
	3rd 248 DP	98.79	99.19	98.39		
	4th 248 DP	98.79	98.79	99.19		

Figures 13, 14, and 15 demonstrate that the use of the filtering techniques achieved comparable performance with higher *β* values, in case of 48, 96, and 248 DPs. For example, in Fig. 13, at − 5 dB, the accuracy increased from 93.83% for *β* = 100 to 93.92% for *β* = 400, while a relatively similar accuracy of 93.66% was obtained with filtering using spectral subtraction. Similarly, in Fig. 14, the accuracy increased from 96.78% for *β* = 100 to 97.14% for *β* = 500, while relatively similar accuracy was obtained using a wiener filter. Likewise, in Fig. 15, the accuracy increased from 98.69% for *β* = 100 to 98.79% for *β* = 400, while relatively similar accuracy was obtained using spectral subtraction.

From the above, it can be concluded that the DWT domain leads to better classification accuracy and watermark integrity compared to the other domains, while the DST resulted in the best-recorded signal quality metrics. However, the DWT also preserved adequate recorded signal quality metrics. Table 7 shows the recorded signal quality metrics at the two ends of the channel at − 5, 0, and + 5 dB SNRs of the AWGN attack using the proposed system using DWT-based SVD watermarking and sequential-based DMpDP having 248 DPs.

**Table 7:** Average quality metrics of recorded signal of the RPS at both ends of the channel in the case of the proposed sequential-based DMpDP for 248 DPs using DWT-based SVD watermarking

Channel SNR	Before transmission through channel				After transmission through channel (recovered at the receiver)			
	SNR	LLR	SD	Cs	SNR	LLR	SD	Cs
− 5 dB	62.91	0.00041	0.014	1	24.43	2.23	1.15	0.998
0 dB	62.91	0.00041	0.014	1	29.44	1.99	0.66	0.999
5 dB	62.91	0.00041	0.014	1	34.33	1.58	0.38	0.999

**Fig. 16:**
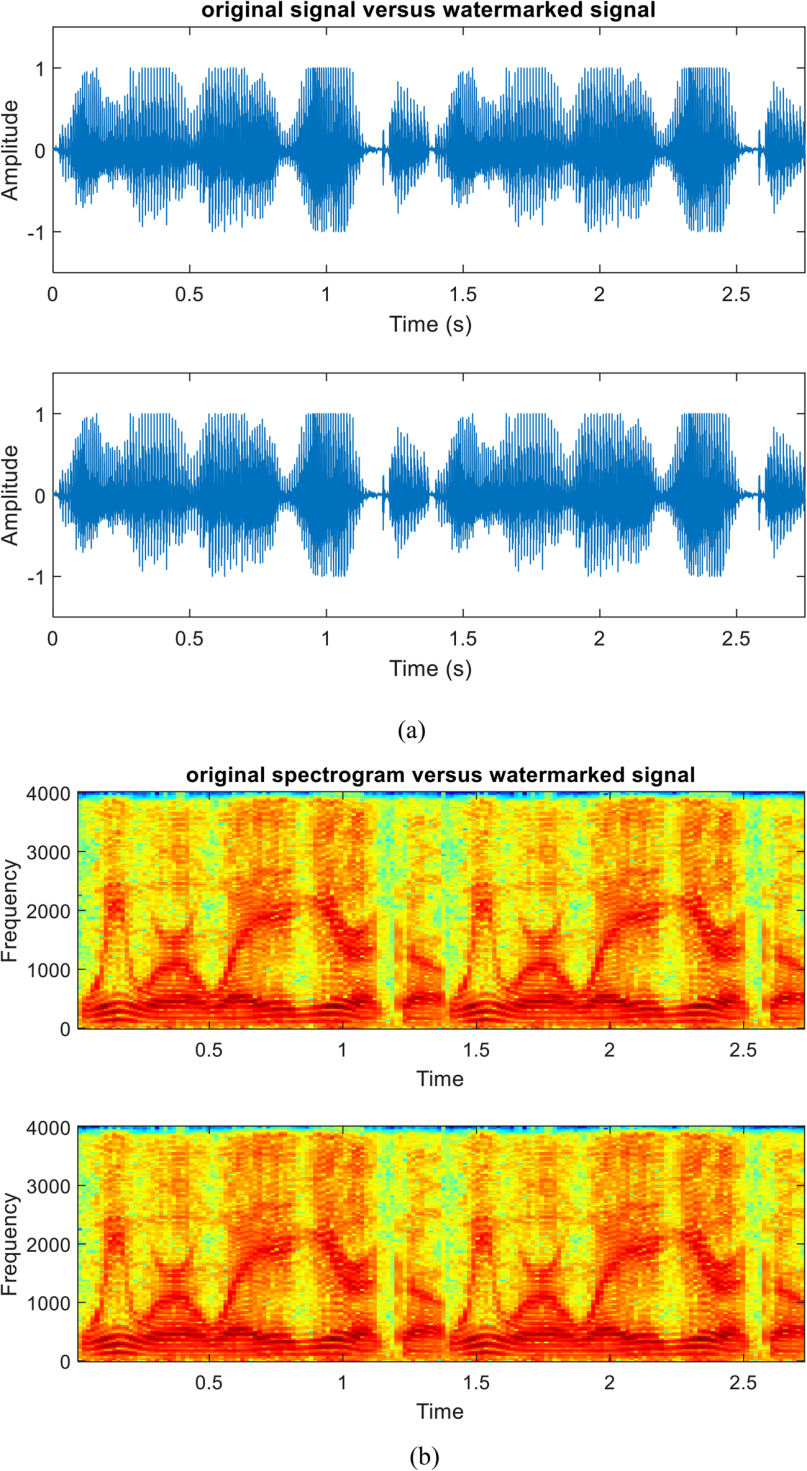

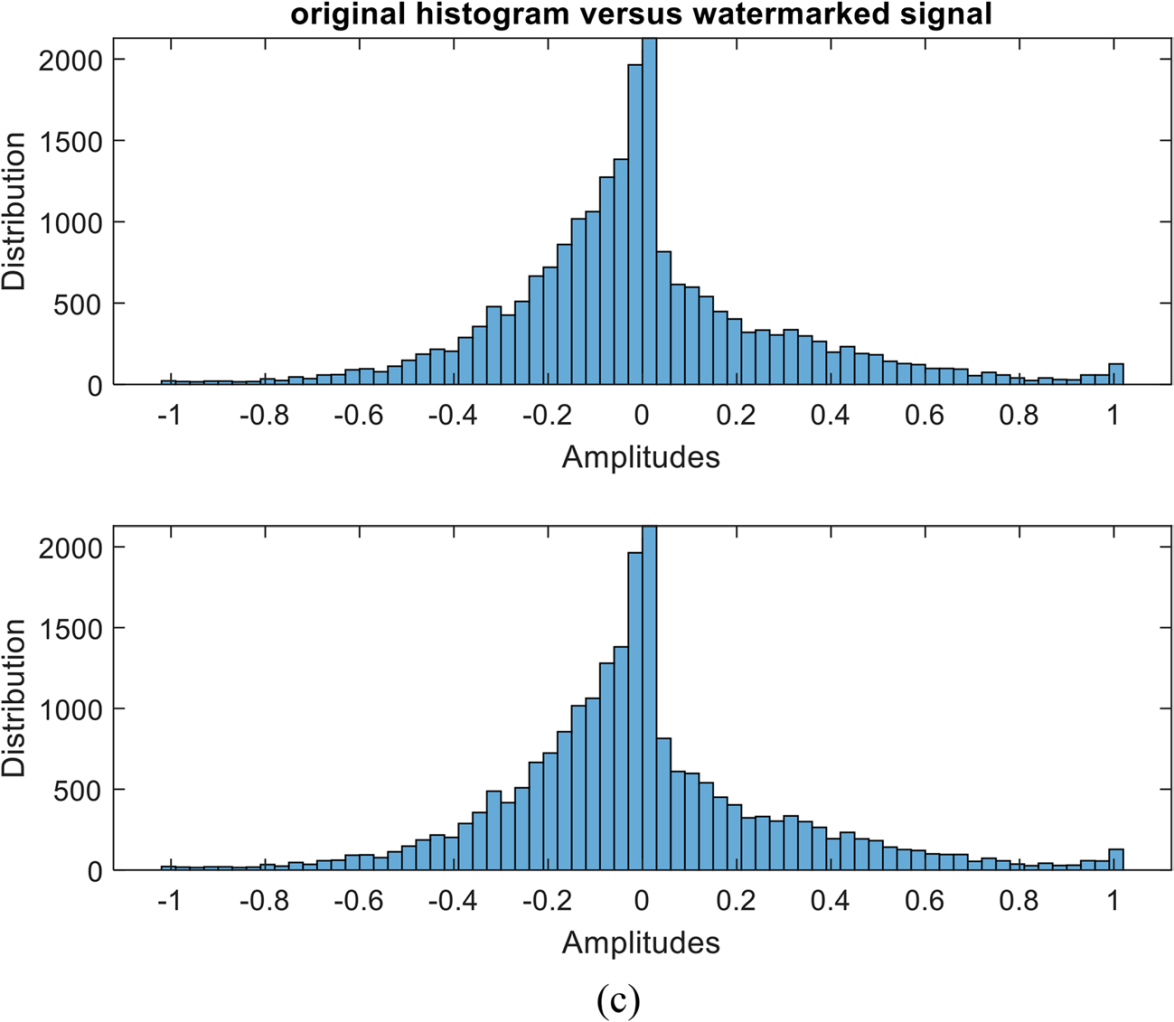
Quality of original and watermarked speech signal after embedding the sequential-based DMpDPs using 248 DPs, where signals in time domain (**a**); signals’ spectrograms (**b**); and signals’ histograms (**c**)

**Fig. 17:**
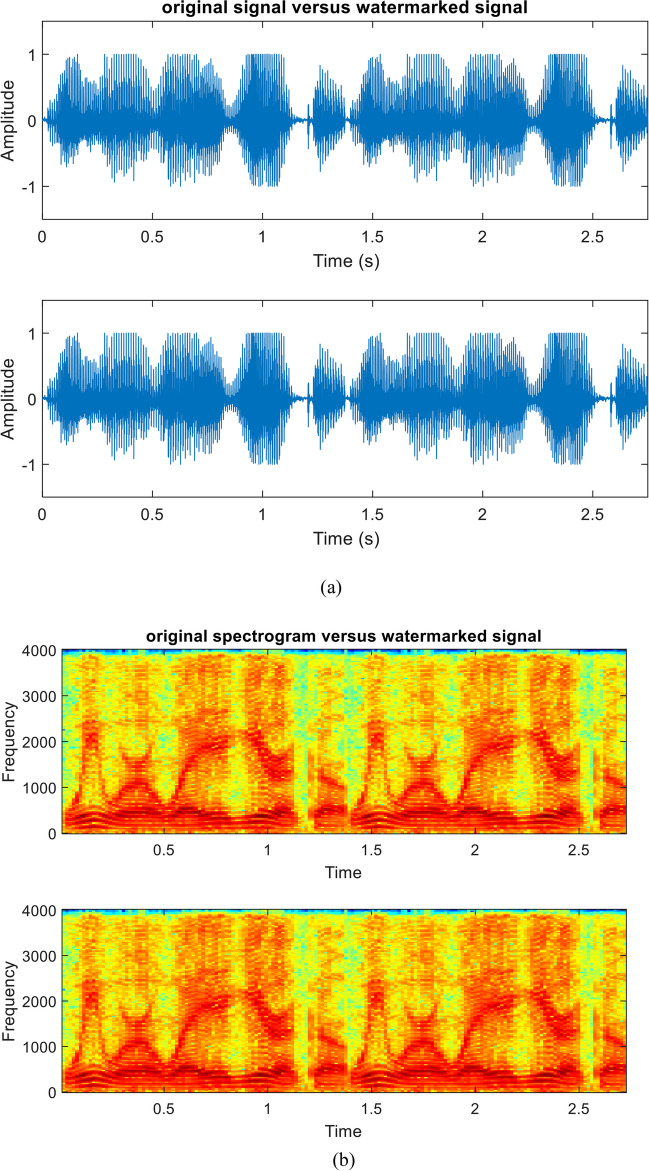

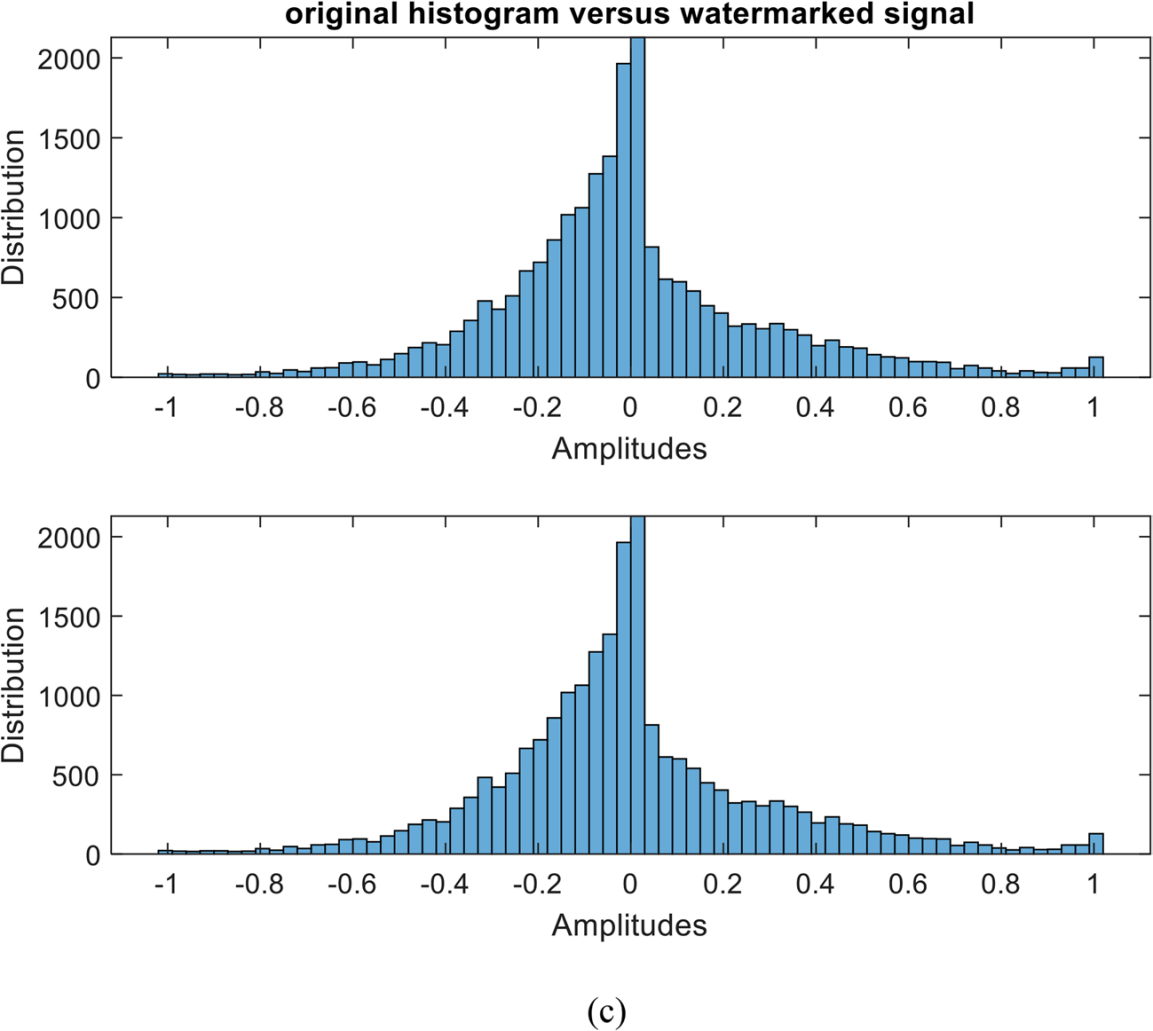
Quality of original and watermarked speech signal after embedding the sequential-based DMpDPs using 96 DPs, where signals in time domain (**a**); signals’ spectrograms (**b**); and signals’ histograms (**c**)

The quality of the original speech signal and the watermarked signal after embedding the sequential-based DMpDP is compared in Figs. 16 and 17 for 248 DPs and 96 DPs, respectively.

In Fig. 16, 12,152 speech samples were required to embed the 248 DPs, which required a speech duration of 1 s; however, in Fig. 17 when embedding the 96 DPs, 4707 samples only were required for the embedding process, which required 0.5 s of speech duration. The frequency of the used speech signal in both cases is 8000 Hz. Also, both figures appear similar because the quality of the speech signal is not affected by the number of DPs in the sequential-based DMpDP.

Table 7 demonstrates the effect of the AWGN attack on the watermarked recorded signal at both ends of the channel. It indicates that the quality of the recovered RPS was average SNR 24.427 at − 5 dB, 2.233 average LLR, 1.148 average SD, and 0.9982 average ${C}_{s}$ after the channel, while for average SNR + 5 dB, better results were obtained, namely 34.325 average SNR, 1.577 average LLR, 0.378 average SD, and 0.9998 average ${C}_{s}$. Moreover, the runtime of the presented system was 5.6 s at the transmitter side, and 4.5 s at the receiver side.

To further verify the importance/impact of our proposed system, Table 8 highlights the advantages of the proposed novel transmission approach in contrast to the traditional transmission of dermoscopy images in respect of the transmission size (in kilobytes), the computational runtime (in seconds), and the required number of recorded samples for the embedding process.

**Table 8:** Comparison of the proposed DMpDP transmission approach against the traditional transmission of the original dermoscopy images

Transmitted data	Transmission size (KB)	Computational runtime (s)	No. of recorded signal samples
Single original dermoscopy image	148.2	0.73	49,400
Multiple dermoscopy images (group of 4 original images)	592.8	1.27	197,600
Proposed one DP-sequential-based DMpDP	0.39	0.041	3038
Proposed 4 DP-sequential-based DMpDP	1.57	0.16	12,152
Proposed 248 DP-sequential-based DMpDP	97.2 (equivalent to 392 bytes per DP)	10.1 (equivalent to 0.041 s per DP)	753,424 (equivalent to 49 samples per DP)

Table 8 demonstrated that the transmission size of a single DP is 392 bytes which results in a total of 97.2 KB for transmitting the proposed 248 DP-sequential-based DMpDP, which is less than the transmission size of a single original dermoscopy image. On the other hand, the required computational time at the transmitter and receiver sides for a single dermoscopy image is approximately 0.73 s, which is 17 times the required time of a single DP. Hence, 17 DPs can be diagnosed using the proposed system during the same time interval that is required for diagnosing the dermoscopy image. Moreover, the dermoscopy image required 49,400 recorded samples for the embedding process. Accordingly, for transmitting 248 dermoscopy images, 12,251,200 recorded audio samples would be required. Conversely, only 1/16 of the required recorded samples are needed for transmitting the same number of dermoscopy images (248 images) using the proposed 248 DP-sequential-based DMpDP representation.

## Discussion

Indeed, the data transmission bandwidths are expanding; however, this is not globally achieved yet, especially that one of the main application areas of telemedicine is in rural areas, where the data transmission bandwidth is still a limitation, and a requirement to be met to achieve reliable service. Moreover, one of the main transmission modes of store-and-forward telemedicine systems is physician-to-physician mode in which a large number of images are transmitted from one physician to another for consultation and giving a second opinion. In such case, a large bandwidth is needed to transmit the large number of high-resolution dermoscopy images, as a single high-resolution dermoscopy image can reach up to several MBs. This is why bandwidth is still a requirement in telemedicine systems, as also mentioned in the following recent papers [37, 38].

The presented work aimed to accomplish a new dermoscopic image representation for telemedicine, namely the Diagnostic Multiple-patient DermoFeature Profiles (DMpDP) by proposing a small unit representing a single dermoscopy image which is called DermoFeature Profile (DP). The proposed new representation can be used in diagnosis due to its application in a CAD system, and it can be embedded in a recorded patient signal (RPS) which provides a compact, secure, efficient, and diagnostic form for representing an integrated patient/s case file for diagnosis at the receiver side. Different forms of DMpDPs were investigated for the proposed DMpDP-based guided SAF teledermoscopy system, namely the horizontally aligned, the diagonal-based, and the sequential-based forms. The effect of the parameters *α* and *β* on the system performance without and with the existence of AWGN attack was also investigated, in addition to the different embedding transform domains.

The results demonstrated that using the horizontally aligned form, a spacing value of 25 samples resulted in adequate RPS quality metrics using the DST-based SVD watermarking. However, it was found that the recorded signal quality severely degrades with the increase in the number of DPs, as reflected by the SD values. Hence, the diagonal-based form and the sequential-based forms were investigated to verify their suitability for achieving wide-scale service by carrying a large number of DPs while preserving both the RPS and the watermark quality. Studying the effect of the number of DPs within both forms revealed that in terms of recorded signal quality metrics, better metrics were obtained in the case of 12 DPs compared to 20 DPs in the case of the diagonal-based form, while for the sequential-based form, the recorded signal quality metrics were relatively constant when the number of DPs increases, as reflected by the SNR and LLR values. Similar to the horizontally aligned form, the DST-based watermarking provided the best-recorded signal quality metrics for the RPS.

The classification accuracy of both diagonal-based and sequential-based forms was evaluated for different values of *α* and *β* in the existence of AWGN attack at SNRs of − 5 dB, 0 dB, and + 5 dB. It was found that increasing *β*, which represents the recorded signal weight, achieved better results compared to decreasing *α*, which represents the watermark weight. However, the maximum obtained accuracies observed using the DWT-based SVD watermarking did not exceed 36% at + 5 dB, which is considered poor performance.

On the other hand, for sequential-based form, the classification accuracies have further increased with the increase in the number of DPs reaching 98.79% for transmitting 248 DPs at − 5 dB at *α* = 1 and *β* = 1000. To reduce *β* to minimize the implementation complexity of the proposed system, different filtering techniques were investigated. It was concluded that at − 5 dB, an accuracy of 98.79% was also obtained using *β* = 400, and relatively equal accuracy was obtained at *β* = 100 using a spectral subtraction filtering technique.

The proposed system reinforced the efforts intending to employ artificial intelligence in transmitting and evaluating wide-scale teledermoscopy systems by achieving a maximum 98.79% diagnostic accuracy using the 248 DPs sequential-based form using DWT-based SVD watermarking under the existence of AWGN attack.

Table 9 compares the performance of the state-of-the-art teledermoscopy systems against our proposed system in terms of diagnostic accuracy, transmission time, transmission size, and the applied security methods. A pilot study was conducted on telediagnosis of a dermoscopic-pathologic procedure for melanocytic skin neoplasms showing 83% diagnostic accuracy [39]. Fabbrocini et al. [40] compared the efficiency of telediagnosis to the face-to-face diagnosis of pink lesions, which revealed that 52% of the studied cases had correct diagnosis compared to 66% in the case of face-to-face consultations. Kroemer et al. [41] captured dermatoscopic and clinical images and evaluated 104 lesions of 80 patients; however, the dermatoscopic images’ quality was poor. The results revealed that 88% diagnosis accuracy for clinical tele-evaluation compared to 82% for teledermoscopy. Moreover, Bandic et al. [42] performed a two-step teledermoscopy process by clinically examining the digital dermoscopic images and then evaluating the same images using the ABCD algorithm [43]. The results established diagnostic accuracy ranged from 81.82 to 90.91%. Ashour et al*.* [44] proposed the compact feature profile (CFP) image that represents the main features of the dermoscopy images, then, embedded the CFP in a speech signal using a DWT-based modified SVD approach. In the presence of AWGN, single-level decomposition with hard thresholding wavelet denoising was applied. The results have demonstrated classification accuracy of 100% to classify malignant melanoma and benign nevus at SNR ranging from 10 to 25 dB, while the classification performance at − 5 dB was 62% with 64% sensitivity. Table 9 includes a comparative study of teledermoscopy systems. However, for fair comparison, studies that applied the same dataset should be considered which was not available. Moreover, this work is a novel method in describing dermoscopic images using their most significant features in a compact, integrated image. Also, in Table 9, we have compared our work to state-of-the-art methods; however, it is mentioned that most works in this domain aimed for using watermarking for patient authentication. Also, the works that applied watermarking for data hiding have not introduced a new form to represent the high-resolution dermoscopy image.

**Table 9:** Comparison with other teledermoscopy systems

Paper	System objective	Targeted classes	Dataset	Transmitted image size	Applied security methods	Diagnostic accuracy	Transmission time (s)
Ferrara et al. (2004)	Telediagnosis using dermoscopic–pathological approach	Melanocytic skin neoplasms	12 cases: 12 dermoscopic images (image per case) and accompanying histological material + clinical images for 9 cases	JPEG images (27–90 KB) + histological material (110–233 KB)	N/A	83% for teledermoscopy 100% for teledermatopathology	N/A
Fabbrocini et al. (2008)	Telediagnosis using dermoscopy and clinical images	Pink lesions	44 lesions: 12 Spitz naevi, 9 melanomas, 8 melanomas in situ, 7 Clark naevi, 2 dermal naevi, 1 spitzoid melanoma, and 5 non-melanocytic	N/A	N/A	52% for SAF teledermoscopy 66% for face-to-face consultation	N/A
Kroemer et al. (2011)	Mobile teledermatology using dermoscopy and clinical images	Benign melanocytic malignant melanocytic, benign non-melanocytic/malignant non-melanocytic	113 skin tumors	N/A	N/A	85% using clinical tele-evaluation 82% using teledermoscopy	N/A
Bandic, Kovacevic, Karabeg, Lazarov, and Opric (2020)	Two-step teledermoscopy process	Benign, and malignant lesions	121 Pigmented skin lesions: 75 benign and 46 malignant	N/A	N/A	For teledermoscopic and histopathologic diagnosis: 90.91% For clinical and teledermoscopic diagnosis: 81.82% For clinical and histopathological diagnosis: 82.64%	N/A
Ashour et al. (2020)	Embedding compact feature profile image in speech signal for teledermoscopy system	Melanoma and nevus	100 dermoscopy images (50 per each class)	N/A	MSVD-based watermarking in DWT domain	100% at SNR ranging from 10 to 25 dB, decreased to 62% at − 5 dB for single CFP	N/A
Our proposed system	Multiple patient DMpDP-based SAF teledermoscopy system	Benign keratoses lesion, basal cell carcinoma, melanoma, and melanocytic nevi	1400 dermoscopy images and recorded patient signal	97.2 KB for 248 DPs equivalent to 248 dermoscopy images	SVD-based watermarking in DWT domain	Using 248 DP-sequential-based DMpDPs: 98.79% under − 5 dB AWGN	10.1 (equivalent to 0.041 s per DP)

*N/A, not available information

From the above, the proposed DMpDP realizes the following benefits:

I. Reduced size of the transmitted image without direct/traditional compression from an average of 148.2 KB for a single dermoscopy image to 97.2 KB for the sequential DMpDP composed of 248 DPs, which carry the significant information of 248 dermoscopy images, which is equivalent to 99.7% reduction in size for the single dermoscopy image,
II. Protected and secure transmission,
III. Reduced time required for transmission due to the size reduction,
IV. Achieved efficient use of the transmission channel bandwidth and network resources, by transmitting a large number of DPs for different patients or different images for the same patient at once in the DMpDP,
V. Generated diagnostic DP from the significant diagnostic features of the skin diseases “in the study” based on a computer-aided diagnosis system to ensure the generating informative image, and
VI. Transmitted patients’ information along with the DP or DMpDP as a recorded patient-information signal, called RPS by embedding the DP or DMpDP in the RPS, and disclosed the medical general description symptoms, patient(s) demographics, the patient(s) history, symptoms and complaints, history of tumors, and the general skin lesion’s description for the patient or patients in the RPS.

In the future, the proposed system can be used to support the developed monitoring systems in [45, 46]. Also, the potential and cost of providing a dual transmission using both DWT and DST domains can be investigated for the receiver to achieve optimal DMpDP and RPS quality, using the DWT-based transmission and the DST-based transmission, respectively.

## Conclusions

In this work, a framework for a diagnostic store and forward system was proposed for the efficient distant screening of skin lesions based on a novel representation of dermoscopy images, namely the Diagnostic Multiple-patient DermoFeature Profile (DMpDP). The DPs are collated for several patients or a single patient over time forming the DMpDP. The generated DMpDP is then embedded in the RPS, which carries the medical patient-related information using DWT-based SVD watermarking. Several attacks may occur during the transmission of the watermarked signal over the teledermoscopy channel, such as the AWGN attack. The filtering technique was applied as an initial stage at the receiver. Subsequently, the embedded DMpDP was extracted to obtain both the DPs and the RPS. The DPs were applied to pre-trained second-order SVM, and the RPS was exploited at the receiver to produce the final decision. Studying the different forms of the proposed DMpDP revealed that the best-recorded signal metrics and the highest classification accuracy were obtained using the sequential-based form. Moreover, the sequential-based DMpDP in the proposed work may carry up to 248 DPs, i.e., patients, achieving 98.79% accuracy using *β* = 100 and spectral subtraction filtering under AWGN attack having SNR of − 5 dB. Therefore, the novel proposed DMpDP represents an innovative compact form that substitutes the need of transmitting high-resolution dermoscopy images through SAF teledermoscopy systems. The transmission of the DMpDP as an embedded watermark in the RPS provides an integrated, compact and secure representation of the patient(s) case file at the diagnostic receiver side.
